# Identification of a novel human memory T-cell population with the characteristics of stem-like chemo-resistance

**DOI:** 10.1080/2162402X.2016.1165376

**Published:** 2016-06-08

**Authors:** Kenji Murata, Tomohide Tsukahara, Makoto Emori, Yuji Shibayama, Emi Mizushima, Hiroshi Matsumiya, Keiji Yamashita, Mitsunori Kaya, Yoshihiko Hirohashi, Takayuki Kanaseki, Terufumi Kubo, Tetsuo Himi, Shingo Ichimiya, Toshihiko Yamashita, Noriyuki Sato, Toshihiko Torigoe

**Affiliations:** a Department of Pathology, Sapporo Medical University School of Medicine, Chuo-ku, Sapporo, Japan; b Department of Orthopaedic Surgery, Sapporo Medical University School of Medicine, Chuo-ku, Sapporo, Japan; c Department of Otolaryngology, Sapporo Medical University School of Medicine, Chuo-ku, Sapporo, Japan; d Department of Human Immunology, Research Institute of Frontier Medicine, Sapporo Medical University School of Medicine, Chuo-ku, Sapporo, Japan

**Keywords:** Adoptive cell transfer; memory T stem cell; peptide vaccine; tumor-associated antigen; viral antigen

## Abstract

High-dose chemotherapy may kill not only tumor cells but also immunocytes, and frequently induces severe lymphocytopenia. On the other hand, patients who recover from the nadir maintain immunity against infection, suggesting the existence of an unknown memory T-cell population with stress resistance, long-living capacity, proliferation and differentiation. Recently, the differentiation system of T-cell memory has been clarified using mouse models. However, the human T-cell memory system has great diversity induced by natural antigens derived from many pathogens and tumor cells throughout life, and profoundly differs from the mouse memory system constructed using artificial antigens and transgenic T cells. Here we report a novel human T-cell memory population, “young memory” T (T_YM_) cells. T_YM_ cells are defined by positive expression of CD73, which represents high aldehyde dehydrogenase 1 (ALDH1) activity and CXCR3 among CD8^+^CD45RA^+^CD62L^+^ T cells. T_YM_ proliferate upon TCR stimulation, with differentiation capacity into T_CM_ and T_EM_ and drug resistance. Moreover, T_YM_ are involved in memory function for viral and tumor-associated antigens in healthy donors and cancer patients, respectively. Regulation of T_YM_ might be very attractive for peptide vaccination, adoptive cell-transfer therapy and hematopoietic stem cell transplantation.

## Introduction

High-dose chemotherapy for musculoskeletal tumors may kill not only tumor cells but also immunocytes, and frequently induces severe lymphocytopenia.^1,2^ On the other hand, patients recovering from the nadir maintain immunity against infection, suggesting the existence of a memory T-cell population with stress resistance, long-living capacity, proliferation and differentiation, that is proposed to be comprised of “memory stem cells”.^3^

Turtle et al. identified human CD8^+^ memory T cells in both the CD62L^+^ central memory (T_CM_) and CD62L^−^ effector memory (T_EM_) cell subsets with the capacity to efflux drugs and survive exposure to chemotherapy.^4^ Gattinoni et al. reported a memory T-cell subset, referred to as stem cell memory T (T_SCM_) cells, possessing long-living capacity, self-renewal, and multi-differentiation into T_CM_, T_EM_, and effector T (T_eff_) cells.^5^ T_SCM_ are defined by naive marker CD45RA^+^CD62L^+^CCR7^+^ and memory marker CD95^+^ and classified between naive and T_CM_ cells. We think that the further identification of novel populations of memory T cells that have superior characteristics of stemness is very important for the fundamental understanding and regulation of the cellular immune system against pathogens and cancer cells.

In the present study, we identified a novel human CD8^+^ T-cell memory population, designated “young” memory (T_YM_) T cells, which have the characteristics of capacity of proliferation, drug resistant and differentiation into T_CM_ and T_EM_, on the basis of the activity of the drug metabolic enzyme, ALDH1.

## Materials and methods

The present study was performed in accordance with the guidelines established by the Declaration of Helsinki, and approved by the Ethics Committee of Sapporo Medical University. The patients, their families, and healthy donors provided informed consent for the use of blood samples in our research.

### Blood and tissue samples

Peripheral blood (PB) was obtained from healthy volunteer donors and from cancer patients and tonsils were obtained from chronic tonsillitis or sleep apnea syndrome patients. Cord blood (CB) was obtained from healthy donors or purchased from Takara (Ohtsu, Japan). Viable cell numbers were determined using a Countess^R^ (Life Technologies).

### Antibodies, peptides, flow cytometry, and cell sorting

Cells were labeled with fluorescent antibodies against CD3, CD8, CD45RA, CD62L, CD73, CXCR3, CXCR4, CD45RO, CD27, CD28, CD95, CD31, CD38, CCR5, CCR7, Bcl-2, PD1, PDL1, CTLA-4, TIM3, LFA-1, ICOS, 7-AAD, and IL-7Rα (BD Biosciences, San Diego, CA), and IL-2β (Biolegend, San Diego, CA). Biotinylated HLA-A*24:02 peptide complex tetramers were constructed by Medical and Biological Laboratories, Co., Ltd. (Nagoya, Japan). Peptides PBF A24.2 (AYRPVSRNI),^6^ Survivin-2B (AYACNTSTL),^7^ HIV env gp160 (RYLRDQQLL), EBV BRLF1 (TYPVLEEMF), and were used in the present study. Cell sorting was performed using a FACS Aria II (BD Bioscience) and data were acquired using a FACS Canto II (BD Bioscience). Collected data were analyzed with BD FACSDiva V6.1.3 (BD Bioscience) and FlowJo software (Tree Star, Ashland, OR).

### ALDEFLUOR assay

The ALDEFLUOR kit (StemCell Technologies, Vancouver, Canada) was used to separate the population with high ALDH1 activity. Cells (5 × 10^6^) were suspended in ALDEFLUOR assay buffer containing an ALDH1 substrate, bodipy-aminoacetaldehyde, at the concentration of 1 μmol/L and incubated for 45 min at 37°C according to the manufacturer's protocol. A specific inhibitor of ALDH1, diethylaminobenzaldehyde (DEAB), was used at 50 mmol/L as a negative control.

### T-cell stimulation

Sorted T lymphocytes were activated by anti-CD3/CD28 conjugated magnetic beads (Invitrogen) at a bead-to-cell ratio of 3:1 and then cultured with recombinant human (rh) IL-7 and rhIL-15 at 15 ng/mL (R&D Systems, Minneapolis, Minnesota, USA). Cytokines and medium were replaced every 3–4 d. Cells were counted at 6–7 d using a Countess (Life Technologies).

### Apoptosis assay

We seeded sorted T lymphocytes into 96-well culture plates at 5 × 10^4^ cells per well for each population of cells. The cells in each population were treated with adriamycin (0.1, 0.5, 1, 5, 10 μM) or carboplatin (62.5, 125, 250, 500, 1,000 μM). After 24 or 48 h of exposure to the chemotherapeutic agents, viability of the cells was determined using an apoptosis assay, which was performed according to the manufacturer's protocol (BD Bioscience).

### RNA preparation and PCR analysis

Total RNAs were extracted from cells using the RNeasyMini Kit (Qiagen, Hilden, Germany). cDNA was synthesized using Superscript III and an oligo(dT) primer (Life Technologies Corp.). Human Multiple Tissue cDNA Panels I and II, and the Human Fetal Multiple Tissue cDNA Panel (Clontech; Mountainview, CA) were used as normal tissue cDNAs. PCR was performed using KOD Dash (TOYOBO, Osaka, Japan) to detect CD73. The primer sequences used were 5′- TGATGAACGCAACAATGGAATC-3′ and 5′- ATGGCAGTGACTTCCTGTGG-3′. The PCR mixture was denatured at 94°C for 2 min, followed by 30 cycles at 94°C for 15 sec, at 58°C for 2 sec, and at 74°C for 30 sec. GAPDH was used as an internal control. Real-time PCR was performed using the StepOne system (Life Technologies Corp.). Primers and probes were designed using the TapMan Gene expression assay (Life Technologies Corp.). Thermal cycling was performed with 40 cycles of 95°C for 1 sec, followed by 60°C for 20 min. Each experiment was done in triplicate and normalized to the GAPDH gene as an internal control.

### Gene expression profiling

RNA from ALDH^high^ cells was labeled with Cy5 dye and RNA from ALDH^low^ cells were labeled with Cy3 dye. The probe mixture was hybridized for 40 h at 65°C using a Human Whole Genome Microarray (G4112F) (Agilent Technologies, Santa Clara, CA). The array was scanned after washing with a G2565BA Microarray Scanner and fluorescent signals were acquired using Feature Extraction software (Agilent Technologies). The average expression ratio of Cy5 to Cy3 was determined per gene. A dye swap experiment was also done to label ALDH^high^ and ALDH^low^ cells with Cy3 and Cy5, respectively. An average ratio of more than 2.0 was determined to indicate differential upregulation in ALDH^high^ cells. The accession number of ArrayExpress is E-MTAB-4080.

### Antigen-specific CTL induction

PBMCs obtained from healthy donors and cancer patients separated into CD8^+^ T-cell subsets and CD8^−^ T cells containing DCs using a FACS Aria II. CD8^−^ cells were incubated for 60 min at room temperature with each peptide. CD8^+^ T-cell subsets (0.5–1 × 10^5^/well) and peptide-pulsed CD8^−^ T cells (1–2 × 10^5^/well) were cocultured in 96-microwell plates in 250 μL of AIM-V (Invitrogen Corp., Carlsbad, CA) with 10% human serum (HS), IL-2 (50 U/mL; a kind gift from Takeda Chemical Industries, Ltd., Osaka Japan), and IL-7 (10 ng/mL). Half of the medium was replaced every 3–4 d with fresh AIM-V containing IL-2 and IL-7. On days 12–14, the cells were subjected to tetramer-based frequency analysis.

## Results

### CD8^+^ALDH^high^ T cells in PBMC contained a population with the characteristics of drug resistance and responsibility for TCR stimulation

**Figure 1.**
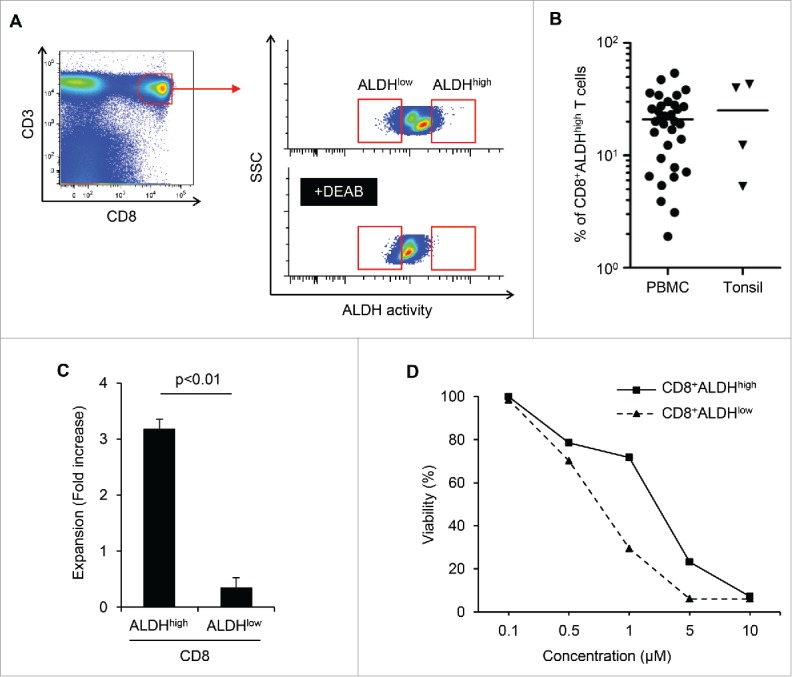
CD8^+^ALDH^high^ T cells in PBMC contained a population with the characteristics of drug resistance and responsibility for TCR stimulation. (A) PBMC demonstrated ALDH activity. FACS analysis of ALDH1 expression in cells and the DEAB control. (B) Proportions of CD8^+^ALDH^high^ T cells in PBMC (n = 33) and tonsils (n = 4). Each point represents data from an individual healthy donor and patient, and bars represent mean. (C) Expansion (measured as fold increases) of ALDH^high^ and ALDH^low^ cells in CD8^+^ T cells activated with bCD3/CD28 and cultured with IL-7 and IL-15 at days 6–7. Data represented mean ± SD of three independent experiments. Statistically significant differences were determined with the Mann–Whitney *U* test. (D) CD8^+^ALDH^high^ T cells were resistant to adriamycin *in vitro*. CD8^+^ALDH^high/low^ cells were cultured in the presence of serially diluted adriamycin and labeled with Annexin V.

We performed ALDEFLUOR assay to detect the population with high activity of ALDH (ALDH^high^) in human lymphocytes. As shown in Fig. 1A, PBMCs and tonsils contained ALDH^high^ populations, although the proportion of ALDH^high^ cells varied. The mean proportions of ALDH^high^ cells among CD8^+^ T cells in PBMCs (n = 33) and tonsils (n = 4) were 21 and 26%, respectively (Fig. 1B). The purity and mRNA expression ALDH of sorted cells was also confirmed (Figs. S1A and B). We then examined the proliferative capacities and drug resistance of CD8^+^ALDH^high^ cells and CD8^+^ALDH^low^ cells. The expansion of CD8^+^ALDH^high^ cells via stimulation with TCR using anti-CD3/CD28 microbeads was significantly higher than that of CD8^+^ALDH^low^ cells (Fig. 1C; Fig. S1C). Moreover, the viability of CD8^+^ALDH^high^ cells in the presence of adriamycin, a standard chemotherapeutic drug for osteosarcoma and synovial sarcoma, was higher than that of CD8^+^ALDH^low^ cells (Fig. 1D; Fig. S1D). We next analyzed surface markers (CD45RA and CD62L) of CD8^+^ALDH^high^ cells and CD8^+^ALDH^low^ cells. It is known that surface markers of conventional naive T cells are CD45RA^+^CD62L^+^, those of central memory T cells are CD45RA^−^CD62L^+^ and those of effector memory T cells are CD45RA^−^CD62L^−^. Interestingly, CD8^+^ALDH^high^ cells contained a higher proportion of CD45RA^+^CD62L^+^ cells than CD8^+^ALDH^low^ cells (Figs. S1E and F). Moreover, the number and proportion of CD45RA^+^CD62L^+^ cells among CD8^+^ADLH^high^ cells was increased by anti-CD3/CD28 stimulation (Fig. S1G). These findings suggested that the drug-resistant CD8^+^ADLH^high^ cells contained a population with high responsibility for TCR stimulation in the CD45RA^+^CD62L^+^ population.

### Identification of CD73 as a marker to represent ALDH1

**Figure 2.**
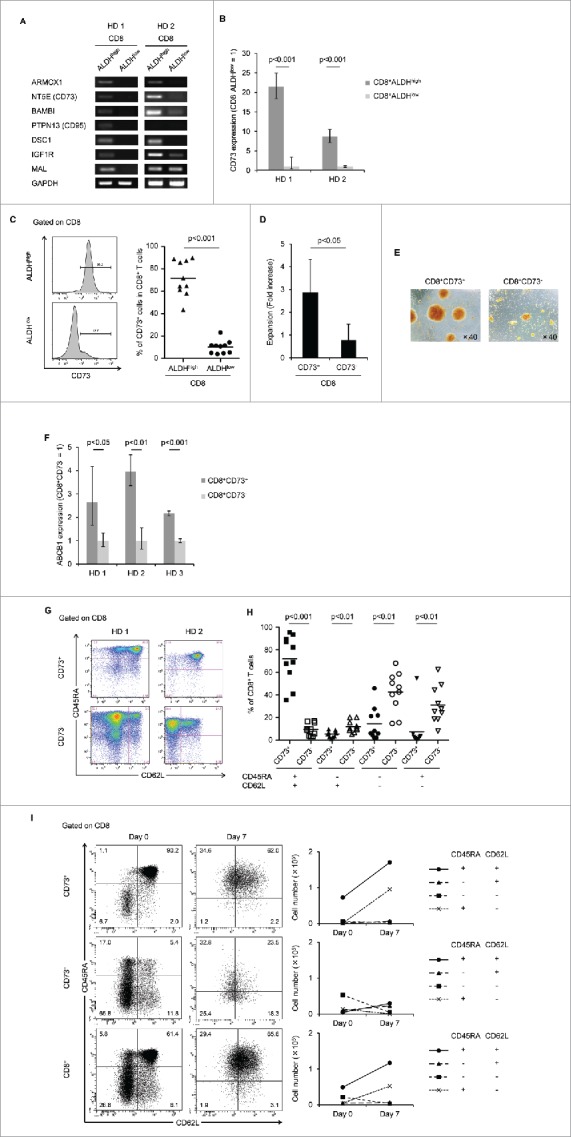
Identification of CD73 as a marker to represent ALDH1. (A) The mRNA expression of seven upregulated genes in two healthy donors (HDs). (B) Expression of CD73 mRNA in CD8^+^ALDH^high^ and CD8^+^ALDH^low^ cells. Data represent mean ± SD. Statistically significant differences were determined with the Mann–Whitney *U* test. (C) FACS analysis of CD73-positive cells in CD8^+^ALDH^high^ and CD8^+^ALDH^low^ cells is shown. Each point represents data from an individual healthy donor, and bars represent mean. Statistically significant differences were determined with the Mann–Whitney *U* test. (D) Expansion (measured as fold increases) of CD73^+^ and CD73^−^ cells in CD8^+^ T cells activated with bCD3/CD28 and cultured with IL-7 and IL-15 at days 6–7. Data represented mean ± SD of six independent experiments. Statistically significant differences were determined with the Mann–Whitney *U* test. (E) The microscopic features of CD8^+^CD73^+^ and CD8^+^CD73^−^ cells reacted with anti-CD3/CD28 microbeads. Data are representative of six independent experiments. (F) Expression of ABCB1 mRNA in CD8^+^CD73^+^ and CD8^+^CD73^−^ cells. Data represent mean ± SD. Statistically significant differences were determined with the Mann–Whitney *U* test. (G) Representative FACS plots of CD45RA and CD62L expression in CD8^+^CD73^+^ and CD8^+^CD73^−^ cells. (H) Proportions of CD73^+^ and CD73^−^ cells in CD8^+^ T-cell subsets from adult PB (n = 10). Each point represents data from an individual healthy donor, and bars represent mean. Statistically significant differences were determined with the Mann–Whitney *U* test. (I) CD8^+^73^+^ and CD8^+^CD73^−^ cells were activated with bCD3/CD28 and cultured with IL-7 and IL-15 for 6–7 d, and then analyzed for expression of CD45RA and CD62L by flow cytometry (left panel). Cell numbers of CD8^+^ T-cell subsets generated from CD8^+^73^+^ and CD8^+^CD73^−^ cells stimulated with bCD3/CD28, IL-7 and IL-15 (right panel). Data are representative of three independent experiments.

We hypothesized that CD8^+^ALDH^high^ cells might contain a novel memory population, and profiled the gene expression of CD8^+^ALDH^high^ cells and CD8^+^ALDH^low^ cells using a cDNA microarray to identify specific markers of a novel memory population. The upregulated genes in CD8^+^ALDH^high^ cells of PBMC (> 2-fold change in expression) are summarized in Extended Data Table S1. We screened membrane-protein-related genes and transcription factors based on information obtained from GeneCards (http://www.genecards.org). We then analyzed the mRNA expression of the top 30 genes in CD8^+^ALDH^high^ and CD8^+^ALDH^low^ cells by RT-PCR (data not shown). Among seven genes, that were upregulated in CD8^+^ALDH^high^ cells, we selected CD73 as a representative marker by checking the reproducibility in another experiment (Figs. 2A and B). Flow cytometric analysis revealed that CD73 expression in CD8^+^ALDH^high^ cells was much higher than in CD8^+^ALDH^low^ cells (Fig. 2C).

We also examined the characteristics of CD8^+^CD73^+^ and CD8^+^CD73^−^ cells. As with the results of ALDEFLUOR assay, CD8^+^CD73^+^ cells showed more proliferative capacity than CD8^+^CD73^−^ cells (Figs. 2D and E). The mRNA expression of the ATP-binding cassette (ABC)-superfamily multidrug efflux protein ABCB1 in CD8^+^CD73^+^ cells was higher than in CD8^+^CD73^−^ cells (Fig. 2F). CD8^+^CD73^+^ cells contained a higher proportion of CD45RA^+^CD62L^+^ cells than CD8^+^CD73^−^ cells (Figs. 2G and H). Moreover, the number of CD45RA^+^CD62L^+^ cells in CD8^+^CD73^+^ cells was increased by TCR stimulation (Fig. 2I).

Thus, CD73 was demonstrated to be a representative marker of ALDH1, and it was hypothesized CD45RA^+^CD62L^+^ cells in CD8^+^CD73^+^ cells might contain a novel memory T-cell population with proliferative capacity and drug resistance.

### Memory T-cell population contained in CD8^+^CD73^+^CD45RA^+^CD62L^+^ cells was close to the naive phenotype

**Figure 3.**
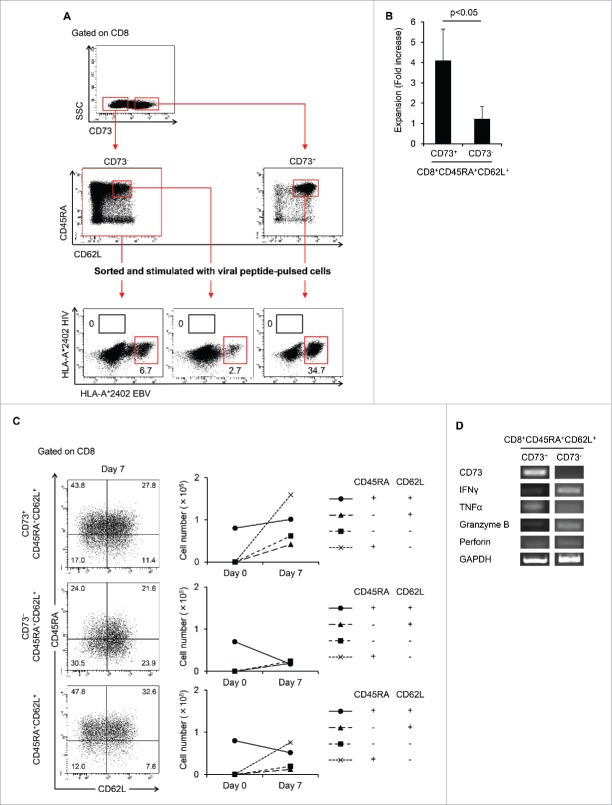
CD8^+^CD73^+^CD45RA^+^CD62L^+^ cells contains a memory cell population. (A) CD8^+^CD73^−^ cells, CD8^+^CD73^−^CD45RA^+^CD62L^+^ cells, and CD8^+^CD73^+^CD45RA^+^CD62L^+^ cells were stimulated with viral peptide-pulsed CD8^−^ T cells and cultured for 12–14 d in the presence of IL-2 and IL-7. The percentage of tetramer^+^ events is shown. Data are representative of six independent experiments. (B) Expansion (measured as fold increases) of CD73^+^ and CD73^−^ cells in CD8^+^CD45RA^+^CD62L^+^ T cells activated with bCD3/CD28 and cultured with IL-7 and IL-15 at days 6–7. Data represented mean ± SD of four independent experiments. Statistically significant differences were determined with the Mann–Whitney *U* test. (C) CD8^+^CD73^+^CD45RA^+^CD62L^+^ and CD8^+^CD73^−^CD45RA^+^CD62L^+^ cells were activated with bCD3/CD28 and cultured with IL-7 and IL-15 for 6–7 d, and then analyzed for expression of CD45RA and CD62L by flow cytometry (left panel). Cell numbers of CD8^+^ T-cell subsets generated from CD8^+^CD73^+^CD45RA^+^CD62L^+^ and CD8^+^CD73^−^CD45RA^+^CD62L^+^ cells stimulated with bCD3/CD28, IL-7 and IL-15 (right panel). Data are representative of four independent experiments. (D) The mRNA expression of cytokine genes in CD8^+^CD73^+^CD45RA^+^CD62L^+^ T cells and CD8^+^CD73^−^CD45RA^+^CD62L^+^ T cells.

To examine whether CD8^+^CD73^+^CD45RA^+^CD62L^+^ cells might contain memory T cells, we investigated whether viral antigen-specific CTL could be induced from those cells. CD8^+^CD73^+^CD45RA^+^CD62L^+^ cells were sorted from HLA-A*24:02 healthy donors, and then stimulated with CD8^−^ T cells pulsed with peptides derived from EBV and HIV and cultured for 12–14 d. CTLs directed to EBV or CMV antigens could be induced from CD8^+^CD73^+^CD45RA^+^CD62L^+^ cells but not to HIV antigen, which was used as a negative control (Fig. 3A). Therefore, we considered that CTLs induced from CD8^+^CD73^+^CD45RA^+^CD62L^+^ cells were derived from memory T cells. On the other hand, CTLs directed to viral antigens could be also induced from CD8^+^CD73^−^CD45RA^+^CD62L^+^ cells.

We then evaluated the proliferative capacity and mRNA expression of cytokine genes in both populations. After TCR stimulation, CD8^+^CD73^+^CD45RA^+^CD62L^+^ cells showed higher proliferative capacity than CD8^+^CD73^−^CD45RA^+^CD62L^+^ cells (Fig. 3B) and could differentiate into other T-cell subsets (Fig. 3C). Furthermore, the number of CD45RA^+^CD62L^+^ cells in CD8^+^CD73^+^ cells increased, whereas the number of CD45RA^+^CD62L^+^ cells among CD8^+^CD73^−^ cells decreased (Fig. 3C). Interestingly, however, the mRNA expression of cytokine genes in the CD8^+^CD73^+^CD45RA^+^CD62L^+^ cells was lower than in CD8^+^CD73^−^CD45RA^+^CD62L^+^ (Fig. 3D). These findings suggested that the novel memory T-cell population in CD8^+^CD73^+^CD45RA^+^CD62L^+^ cells might be close to the naive phenotype and that CD8^+^CD73^−^CD45RA^+^CD62L^+^ cells might contain a memory population close to the conventional memory phenotype like T_CM_ and T_EM_.

### CXCR3 divided CD8^+^CD73^+^CD45RA^+^CD62L^+^ cells into the “pure” naive phenotype and novel memory phenotype

**Figure 4.**
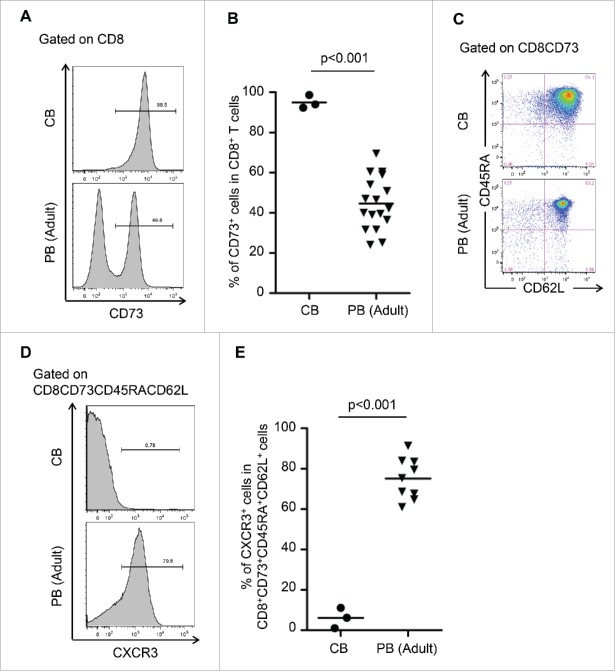
CXCR3 divided CD8^+^CD73^+^CD45RA^+^CD62L^+^ cells into pure naive and novel memory phenotypes. (A) Representative FACS plots of CD73 expression on CD8^+^ T cells in cord blood (CB) and adult peripheral blood (PB). (B) Proportion of CD8^+^ T cells expressing CD73 in cord blood and adult peripheral blood. Each point represents data from an individual healthy donor, and bars represent mean. Statistically significant differences were determined with the Mann–Whitney *U* test. (C) Representative FACS plots of CD45RA and CD62L expression in CD8^+^CD73^+^ cells on cord blood and adult peripheral blood cells. Data are representative of three independent experiments (CB) and ten independent experiments (PB). (D) Representative FACS plots of CXCR3 expression on CD8^+^CD73^+^CD45RA^+^CD62L^+^ T cells in cord blood and adult peripheral blood. (E) Proportion of CD8^+^CD73^+^CD45RA^+^CD62L^+^ T cells expressing CXCR3 in cord blood and adult peripheral blood. Each point represents data from an individual healthy donor, and bars represent mean. Statistically significant differences were determined with the Mann–Whitney *U* test.

We investigated surface markers of CB cells, which are known to be pure naive.^8,9^ Interestingly, flow cytometric analysis revealed that the expression of CD73 among CD8^+^ cells in CB was significantly higher than in adult PB (Figs. 4A and B), and CB also contained more than 90% of CD73^+^CD45RA^+^CD62L^+^ cells among CD8^+^ cells (Fig. 4C). Therefore, CD8^+^CD73^+^CD45RA^+^CD62L^+^ cells comprise a “pure” naive population and we found that these cells in PB contained both a pure naive population and novel memory population. To divide further CD8^+^CD73^+^CD45RA^+^CD62L^+^ cells into the two populations, we selected CXCR3, which is known as a memory marker ^10,11^ and is upregulated in CD8^+^ALDH^high^ cells (1.41-fold change in expression). Flow cytometric analysis revealed that expression of CXCR3 among CD8^+^CD73^+^CD45RA^+^CD62L^+^ cells in CB was significantly lower than in PB (Figs. 4D and E). Based on these findings, we successfully defined CD8^+^CD73^+^CD45RA^+^CD62L^+^CXCR3^−^ cells as the pure naive population and CD8^+^CD73^+^CD45RA^+^CD62L^+^CXCR3^+^ cells as the novel memory population.

### Novel memory T-cell population with the features of drug resistance, responsibility for TCR stimulation, and antigen specificity: Young memory T (T_YM_) cells

**Figure 5.**
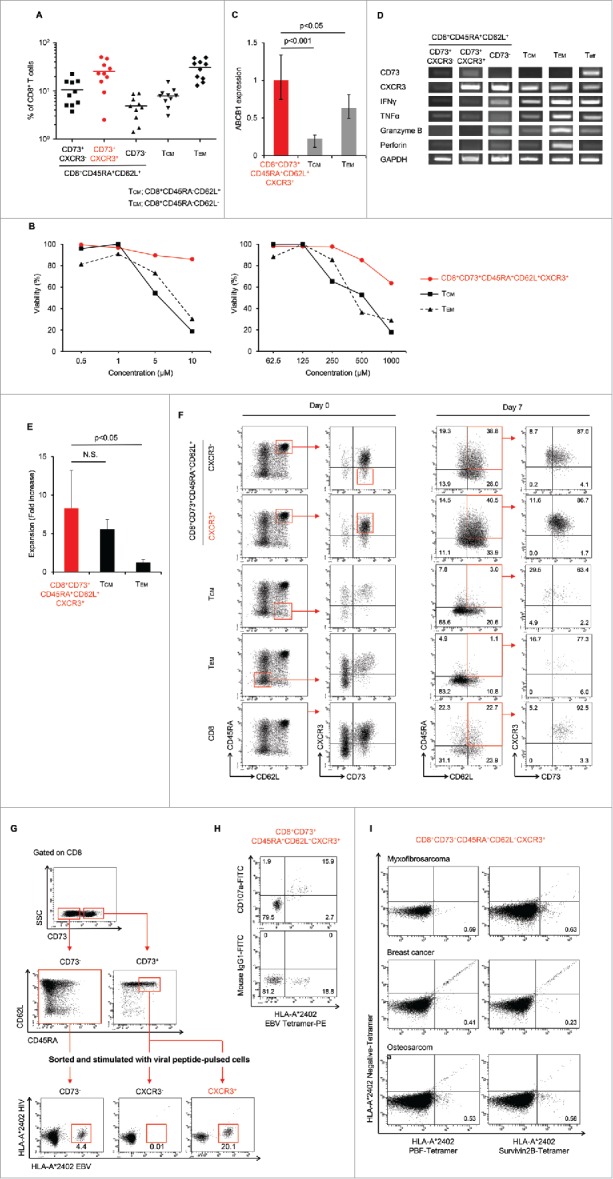
Identification of a novel memory T cell population; young memory T cells (TYM).(A) Percentages of CD8+ T cell subsets in adult peripheral blood (n=10). Each point represents data from an individual healthy donor, and bars represent mean.(B) CD8+CD73+CD45RA+CD62L+CXCR3+ T cells and known memory CD8+ T cell subsets were cultured in the presence of serially diluted Adriamycin (left) and carboplatin (right), and labeled with Annexin V.(C) Expression of ABCB1 mRNA in CD8+CD73+CD45RA+CD62L+CXCR3+ T cells and known memory CD8+ T cell subsets. Bars represent mean ± SEM. Data represent mean ± SD. Statistically significant differences were determined with the Mann–Whitney U test.(D) The mRNA expression of cytokine genes in CD8+ T cell subsets.(E) Expansion of CD8+CD73+CD45RA+CD62L+CXCR3+ T cells and known memory CD8+ T cell subsets activated with bCD3/CD28 and cultured with IL-7 and IL-15 at days 6-7. Data represented mean ± SD of five independent experiments. Statistically significant differences were determined with the Mann–Whitney U test.(F) Differentiation of T cells upon TCR stimulation using bCD3/CD28, IL-7 and IL-15 at Day 7. Data are representative of three independent experiments.(G) CD8+CD73- T cells, CD8+CD73+CD45RA+CD62L+CXCR3- T cells and CD8+CD73+CD45RA+CD62L+CXCR3+ T cells were stimulated with CD8- T cells pulsed with epitopes from EBV and HIV, and cultured for 12-14 days in the presence of IL-2 and IL-7. The percentage of tetramer+ events is shown. Data are representative of six independent experiments.(H) Detection of CD107a exposed on the cell surface after antigen stimulation. HLA-A*24:02 restricted CTL were stimulated using an epitope peptide in the presence of an FITC-labeled CD107a monoclonal antibody and cultured for 5 hours at 37°C. After culture, the cells were stained with HLA-A*24:02 EBV tetramer-PE.(I) CD8+CD73+CD45RA+CD62L+CXCR3+ T cells were stimulated with CD8- T cells pulsed with epitopes from PBF or survivin-2B, and cultured for 12-14 days in the presence of IL-2 and IL-7. The percentage of tetramer+ events is shown.

We evaluated the characteristics of CD8^+^CD73^+^CD45RA^+^CD62L^+^CXCR3^+^ cells and the conventional memory cell population. The mean proportion of CD8^+^CD73^+^CD45RA^+^CD62L^+^CXCR3^+^ cells among all CD8^+^ cells was 25% (Fig. 5A). The viabilities of CD8^+^CD73^+^CD45RA^+^CD62L^+^CXCR3^+^ cells in the presence of adriamycin or carboplatin were higher than those of T_CM_ and T_EM_ (Fig. 5B). Moreover, the ABCB1 mRNA expression in CD8^+^CD73^+^CD45RA^+^CD62L^+^CXCR3^+^ cells was higher than in T_CM_ and T_EM_ (Fig. 5C). In addition, the mRNA expression of cytokine genes in CD8^+^CD73^+^CD45RA^+^CD62L^+^CXCR3^+^ cells was similar to T_CM_, contrasting with the “pure” naive (CD8^+^CD73^+^CD45RA^+^CD62L^+^CXCR3^−^) cells (Fig. 5D). The proliferative capacity of CD8^+^CD73^+^CD45RA^+^CD62L^+^CXCR3^+^ cells was higher than that of T_EM_ and comparable to that of T_CM_ (Fig. 5E). Moreover, CD8^+^CD73^+^CD45RA^+^CD62L^+^CXCR3^+^ T cells could differentiate into T_CM_ and T_EM_ upon TCR stimulation (Fig. 5F; Fig. S2). These results suggested that CD8^+^CD73^+^CD45RA^+^CD62L^+^CXCR3^+^ T cells had higher drug resistance and proliferative capacity than conventional memory cell populations.

Next, we stimulated CXCR3^+^ and CXCR3^−^ cells among CD8^+^CD73^+^CD45RA^+^CD62L^+^ cells with viral peptides presented by HLA-A*24:02. As expected, viral antigen EBV-specific CTLs could be induced from CD8^+^CD73^+^CD45RA^+^CD62L^+^CXCR3^+^ cells but not from CD8^+^CD73^+^CD45RA^+^CD62L^+^CXCR3^−^ cells (Fig. 5G). As a result, CD8^+^CD73^+^CD45RA^+^CD62L^+^CXCR3^−^ cells were identified as a “pure” naive T-cell population. We also detected CD107a exposed on the cell surface after antigen stimulation by CD107 mobilization assay. CD107a expression in CTLs induced from CD8^+^CD73^+^CD45RA^+^CD62L^+^ CXCR3^+^ cells was verified by flow cytometry (Fig. 5H). Furthermore, CTLs directed to tumor-associated antigens survivin2B and PBF could be induced from CD8^+^CD73^+^CD45RA^+^CD62L^+^CXCR3^+^ cells of HLA-A*24:02+ cancer patients (Fig. 5I). We designated the CD8^+^CD73^+^CD45RA^+^CD62L^+^CXCR3^+^ cells, novel memory T cells close to the naive phenotype, “young memory” T (T_YM_) cells.

### Young memory T cells were closer to the naive phenotype than stem cell memory T cells

**Figure 6.**
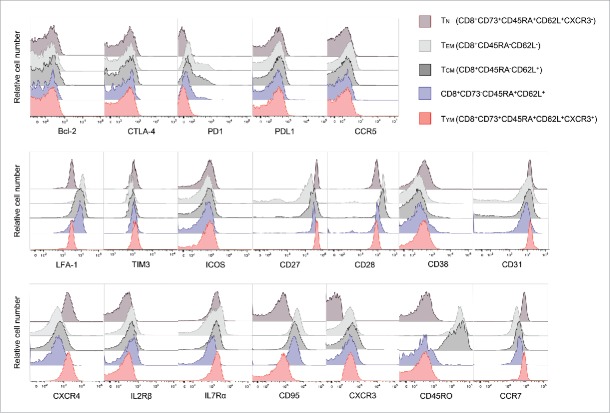
T_YM_ cells are a novel memory population close to the pure naive phenotype. Flow cytometric analysis of expression of cell surface molecules. Overlaid histogram plots show expression levels of the given molecules in CD8^+^ T-cell subsets.

We analyzed the phenotypes of CD8^+^ T-cell subsets including CD8^+^CD73^−^CD45RA^+^CD62L^+^ cells. T_YM_ and T_N_ cells could be distinguished from each other on the basis of the expression of CXCR3, but otherwise had similar surface phenotypes (Fig. 6).^12^ On the other hand, the surface phenotype of CD8^+^CD73^−^CD45RA^+^CD62L^+^ cells was similar to those of T_CM_ and T_EM_. Notably, these cells had higher expression of CD95, known as a molecule associated with memory T cells, than T_YM_cells.

## Discussion

In the present study, we demonstrated that: (i) ALDH^high^ cells, which were stress resistant and highly proliferative upon TCR stimulation, existed among the CD8^+^CD45RA^+^CD62L^+^ population, (ii) CD73 was overexpressed in ALDH^high^ cells and representative of ALDH activity, (iii) CD8^+^CD73^+^CD45RA^+^CD62L^+^ cells contained both viral antigen-specific memory and naive phenotypes, (iv) CD8^+^CD73^+^CD45RA^+^CD62L^+^ cells were successfully divided into CXCR3^+^ (a novel “young memory”; T_YM_) and CXCR3^−^ (“pure” naive) cells, (v) T_YM_ had proliferation, drug resistance, could differentiate into T_CM_ and T_EM_ and had memory directed to both virus and tumor-associated antigens, and (vi) T_YM_ barely expressed CD95, as the same as the naive cells. These findings suggested the hypothesis that T_YM_ were novel memory T cells located closer to naive cells than T_SCM_ in the T-cell differentiation linage.

CD73, also known as ecto-5′-nucleotidase (ecto-5′-NT, _EC_ 3.1.3.5), is a glycosyl-phosphatidylinositol (GPI)-linked 70-kDa cell surface enzyme found in most tissues.^13,14^ Originally defined as a lymphocyte differentiation antigen, CD73 is thought to function as a cosignaling molecule on T lymphocytes ^15^ and as an adhesion molecule that is important for lymphocyte binding to endothelium.^13,16^ It is upregulated in naive T cells,^17^ B cells,^18^ in CD4^+^CD25^+^Foxp3^+^ regulatory T cells,^19,20^ and in various human carcinomas,^21^ and suppresses immune responses by producing extracellular adenosine.^22^ Recently, inhibition of CD73 was reported to reduce tumorigenesis and metastasis, as well as enhance the potency of T-cell-directed therapies.^21,23^ Depletion of CD73^+^ T cells might reduce the number of regulatory T cells and amount of extracellular adnosine, and contribute to a good prognosis for a short period. However, depletion of CD73^+^ cells simultaneously reduced T_YM_ and it might inevitably impair cellular immunity against cancer over the long term.

CXCR3 is a chemokine receptor that is highly expressed on effector T cells and plays an important role in T-cell trafficking and function.^24,25^ It is rapidly induced on naive cells following activation and preferentially remains highly expressed on effector CD8^+^ T cells.^26,27^, CXCR3^+^ was expressed on from 60–90% of CD8^+^ memory T cells,^10,11^ but not on naive cells of adult PB and neonatal CB. Using the combination of CD73 highly expressed on ALDH^high^ cells and CXCR3 as a memory cell marker, we successfully isolated a novel memory cell population, T_YM_ cells, from CD8^+^CD45RA^+^CD62L^+^CCR7^+^ cells of adult PB.

Turtle et al. identified a drug-resistant T-cell population, CD8^+^CD161^hi^IL-18R^hi^ T cells, among both the T_CM_ and T_EM_ subsets. In the present study, T_YM_ cells were not included in T_CM_ and T_EM_, but in CD45RA^+^CD62L^+^ cells. Moreover, ALDH^high^ cells did not contain the CD8^+^CD161^hi^IL-18R^hi^ T cells.^28^ Therefore, we considered that T_YM_ cells were different from CD8^+^CD161^hi^IL-18R^hi^ T cells.

Gattinoni et al. proposed the idea of T_SCM_, which conferred self-renewal capacity, proliferation and multi-differentiation on the T-cell linage.^5^ T_SCM_ were defined by expression of the memory cell marker CD95 (Fas) among the CD8^+^CD45RA^+^CD62L^+^CCR7^+^ population, and characterized differently from classical memory T_CM_ and T_EM_cells. In contrast with T_SCM_, T_YM_ barely expressed CD95, suggesting that T_YM_ was the different population from T_SCM_. However, a very small population of T_SCM_ in T_YM_ should be considered (Fig. S3C). Therefore, we additionally performed experiments using CD95-depleted T_YM_ (Figs. S3A–E) and found that the characteristics of CD95-depleted T_YM_ regarding proliferation, chemo-resistance, differentiation, and antigen-specific memory were compatible to those of T_YM_.

The comparison of the functions of T_YM_ and T_SCM_ is important. However, it was very hard because of the difficulty in isolating a small population of T_SCM_ (less than 1% in peripheral CD8+ T cells) with certain viability. Instead of this, we are planning to perform RNAseq transcriptome analysis using these populations.

We characterized the memory phenotype of T_YM_ on the basis of CTL induction capacity using epitope peptides derived from virus and tumor-associated antigens. Peptide-specific CTL could be induced from T_YM_, CD73^−^ cells including T_CM_ and T_EM_, but not from CD73^+^CD45RA^+^CD62L^+^CXCR3^−^ (pure naive) cells. This observation suggested that T_YM_ had a distinct memory phenotype not shared by pure naive T cells. Considering the proliferative capacity and drug resistance, T_YM_ might play an important role in the recovery of long-lasting immune surveillance against viral pathogens and tumor cells after the nadir during intensive myelosuppressive chemotherapy, especially in patients with bone and soft tissue sarcomas. Recently, Maraca et al. reported the existence of a naive-like CD8^+^ T-cell response for more than 25 y after vaccination for yellow fever.^29^ Interestingly, the specific T cells resembled T_SCM_. These observations could support the idea that T_SCM_ play a role in the maintenance of long-lasting immunity against pathogens. It is presumed that T_YM_ also contribute to maintain permanent immunity, like T_SCM_.

Furthermore, the idea of T_YM_ could shed light on the great diversity of naive to restricted pure naive T-cell populations. In haematopietic stem cell transplantation (HSCT) for patients with leukemia, the use of donor-derived naive T cells might lead to the development of chronic graft-versus-host disease (GVHD) and depletion of naive T cells from allografts has been recommended.^30^ Preservation of donor T_YM_ in HSCT could contribute to the reconstruction of long-lasting T-cell memory with large diversity in the recipient. Obviously, considering the long-living capacity of T_YM_, it would be very attractive to regulate T_YM_ directed to tumor-associated antigens in the clinical setting of peptide vaccination and adoptive cell-transfer therapies.

In conclusion, here we report on a novel human T-cell memory population, “young memory” T (T_YM_) cells. T_YM_ cells are defined by CD73^+^CXCR3^+^CD45RA^+^CD62L^+^ among CD8^+^ cells and have the capacity to proliferate, drug resistance, and the ability to differentiate into T_CM_ and T_EM_ cells. Regulation of T_YM_ might be useful for peptide vaccination, adoptive cell transfer therapies, and HSCT.

## Supplementary Material

KONI_A_1165376_supplemental_material.zip
