# Supplementary material for: Identification of a novel human memory T-cell population with the characteristics of stem-like chemo-resistance
Source: Oncoimmunology. 2016 Jun 8;5(6):e1165376. doi: 10.1080/2162402X.2016.1165376 (PMC4938359; doi:10.1080/2162402X.2016.1165376)
Supplement: KONI_A_1165376_supplemental_material.zip [file koni-05-06-1165376-s001.zip › KONI_A_1165376_s03.pdf]

Extended Data Table 1.Upregulated genes in CD8+ALDH^{high} cells of PBMC (>2-fold change in expression) in cDNA microarray data.

	GeneName	SystematicName	Description	Cy3/Cy5	Cy5/Cy3
1	AA496144	AA496144	gb zv51g10.r1 Soares_testis_NHT Homo sapiens cDNA clone IMAGE:757218 5', mRNA sequence [AA496144]	2.21	2.41
2	ACCN2	NM_020039	Homo sapiens amiloride-sensitive cation channel 2, neuronal (ACCN2), transcript variant 1, mRNA [NM_020039]	2.89	2.09
3	ACTN1	NM_001102	Homo sapiens actinin, alpha 1 (ACTN1), transcript variant 2, mRNA [NM_001102]	2.41	2.72
4	ACVR1C	NM_145259	Homo sapiens activin A receptor, type IC (ACVR1C), transcript variant 1, mRNA [NM_145259]	2.46	2.86
5	ADM	NM_001124	Homo sapiens adrenomedullin (ADM), mRNA [NM_001124]	2.19	4.32
6	AGPAT9	NM_032717	Homo sapiens 1-acylglycerol-3-phosphate O-acyltransferase 9 (AGPAT9), mRNA [NM_032717]	2.65	3.04
7	AGPHD1	NM_001013619	Homo sapiens aminoglycoside phosphotransferase domain containing 1 (AGPHD1), transcript variant 1, mRNA [NM_001013619]	2.82	2.64
8	ALOX5	NM_000698	Homo sapiens arachidonate 5-lipoxygenase (ALOX5), mRNA [NM_000698]	2.59	2.68
9	ALPK1	NM_025144	Homo sapiens alpha-kinase 1 (ALPK1), transcript variant 1, mRNA [NM_025144]	2.04	2.73
10	ARC	NM_015193	Homo sapiens activity-regulated cytoskeleton-associated protein (ARC), mRNA [NM_015193]	2.30	2.27
11	AREG	NM_001657	Homo sapiens amphiregulin (AREG), mRNA [NM_001657]	2.78	2.19
12	ARHGEF4	NM_032995	Homo sapiens Rho guanine nucleotide exchange factor (GEF) 4 (ARHGEF4), transcript variant 2, mRNA [NM_032995]	2.24	2.30
13	ARL4A	NM_005738	Homo sapiens ADP-ribosylation factor-like 4A (ARL4A), transcript variant 1, mRNA [NM_005738]	2.32	2.38
14	ARL4D	NM_001661	Homo sapiens ADP-ribosylation factor-like 4D (ARL4D), mRNA [NM_001661]	2.59	2.39
15	ARMCX1	NM_016608	Homo sapiens armadillo repeat containing, X-linked 1 (ARMCX1), mRNA [NM_016608]	3.31	11.23
16	ASB13	NM_024701	Homo sapiens ankyrin repeat and SOCS box-containing 13 (ASB13), transcript variant 1, mRNA [NM_024701]	2.42	2.54
17	ATP8A2	NM_016529	Homo sapiens ATPase, aminophospholipid transporter, class I, type 8A, member 2 (ATP8A2), mRNA [NM_016529]	2.60	2.06
18	AXIN2	NM_004655	Homo sapiens axin 2 (AXIN2), mRNA [NM_004655]	2.26	2.34
19	BAMBI	NM_012342	Homo sapiens BMP and activin membrane-bound inhibitor homolog (Xenopus laevis) (BAMBI), mRNA [NM_012342]	4.31	4.08
20	C11orf74	NM_138787	Homo sapiens chromosome 11 open reading frame 74 (C11orf74), mRNA [NM_138787]	3.83	4.89
21	C11orf96	NM_001145033	Homo sapiens chromosome 11 open reading frame 96 (C11orf96), mRNA [NM_001145033]	2.96	2.81
22	C13orf15	NM_014059	Homo sapiens chromosome 13 open reading frame 15 (C13orf15), mRNA [NM_014059]	2.03	2.26
23	C1orf172	NM_152365	Homo sapiens chromosome 1 open reading frame 172 (C1orf172), mRNA [NM_152365]	2.69	2.52
24	C1orf87	NM_152377	Homo sapiens chromosome 1 open reading frame 87 (C1orf87), mRNA [NM_152377]	2.59	2.95
25	C2orf40	NM_032411	Homo sapiens chromosome 2 open reading frame 40 (C2orf40), mRNA [NM_032411]	3.96	3.85
26	C2orf89	NM_001080824	Homo sapiens chromosome 2 open reading frame 89 (C2orf89), mRNA [NM_001080824]	2.65	2.31
27	C3orf59	NM_178496	Homo sapiens chromosome 3 open reading frame 59 (C3orf59), mRNA [NM_178496]	3.00	4.89
28	C6orf105	NM_032744	Homo sapiens chromosome 6 open reading frame 105 (C6orf105), transcript variant 2, mRNA [NM_032744]	2.36	2.32
29	CA6	NM_001215	Homo sapiens carbonic anhydrase VI (CA6), mRNA [NM_001215]	3.57	4.61
30	CAMTA1	NM_015215	Homo sapiens calmodulin binding transcription activator 1 (CAMTA1), transcript variant 1, mRNA [NM_015215]	2.09	2.64
31	CCR9	NM_031200	Homo sapiens chemokine (C-C motif) receptor 9 (CCR9), transcript variant A, mRNA [NM_031200]	3.05	2.21
32	CD248	NM_020404	Homo sapiens CD248 molecule, endosialin (CD248), mRNA [NM_020404]	2.47	2.49
33	CDKN1C	NM_000076	Homo sapiens cyclin-dependent kinase inhibitor 1C (p57, Kip2) (CDKN1C), transcript variant 1, mRNA [NM_000076]	2.22	2.48
34	CITED4	NM_133467	Homo sapiens Cbp/p300-interacting transactivator, with Glu/Asp-rich carboxy-terminal domain, 4 (CITED4), mRNA [NM_133467]	2.69	2.45
35	CLEC11A	NM_002975	Homo sapiens C-type lectin domain family 11, member A (CLEC11A), mRNA [NM_002975]	2.67	3.72
36	CLN5	NM_006493	Homo sapiens ceroid-lipofuscinosis, neuronal 5 (CLN5), mRNA [NM_006493]	2.22	2.51
37	CMTM8	NM_178868	Homo sapiens CKLF-like MARVEL transmembrane domain containing 8 (CMTM8), mRNA [NM_178868]	2.11	2.66
38	CNN3	NM_001839	Homo sapiens calponin 3, acidic (CNN3), mRNA [NM_001839]	2.86	6.99

39	COL6A1	NM_001848	Homo sapiens collagen, type VI, alpha 1 (COL6A1), mRNA [NM_001848]	2.27	2.10
40	CPAMD8	NM_015692	Homo sapiens C3 and PZP-like, alpha-2-macroglobulin domain containing 8 (CPAMD8), mRNA [NM_015692]	3.14	2.80
41	CR2	NM_001006658	Homo sapiens complement component (3d/Epstein Barr virus) receptor 2 (CR2), transcript variant 1, mRNA [NM_001006658]	5.04	5.32
42	CRB3	NM_139161	Homo sapiens crumbs homolog 3 (Drosophila) (CRB3), transcript variant 2, mRNA [NM_139161]	2.65	3.55
43	CTH	NM_001902	Homo sapiens cystathionase (cystathionine gamma-lyase) (CTH), transcript variant 1, mRNA [NM_001902]	2.07	2.06
44	DFNB59	NM_001042702	Homo sapiens deafness, autosomal recessive 59 (DFNB59), mRNA [NM_001042702]	2.46	2.51
45	DISC1	NM_018662	Homo sapiens disrupted in schizophrenia 1 (DISC1), transcript variant L, mRNA [NM_018662]	2.16	2.09
46	DLL1	NM_005618	Homo sapiens delta-like 1 (Drosophila) (DLL1), mRNA [NM_005618]	2.91	4.29
47	DMRTC1	NM_033053	Homo sapiens DMRT-like family C1 (DMRTC1), mRNA [NM_033053]	2.49	2.41
48	DPYSL4	NM_006426	Homo sapiens dihydropyrimidinase-like 4 (DPYSL4), mRNA [NM_006426]	3.00	3.32
49	DSC1	NM_004948	Homo sapiens desmocollin 1 (DSC1), transcript variant Dsc1b, mRNA [NM_004948]	3.20	2.58
50	DSEL	NM_032160	Homo sapiens dermatan sulfate epimerase-like (DSEL), mRNA [NM_032160]	2.29	2.17
51	EDA	NM_001399	Homo sapiens ectodysplasin A (EDA), transcript variant 1, mRNA [NM_001399]	2.64	3.87
52	EDAR	NM_022336	Homo sapiens ectodysplasin A receptor (EDAR), mRNA [NM_022336]	2.22	2.88
53	EGR1	NM_001964	Homo sapiens early growth response 1 (EGR1), mRNA [NM_001964]	2.69	2.36
54	EGR3	NM_004430	Homo sapiens early growth response 3 (EGR3), transcript variant 1, mRNA [NM_004430]	5.11	14.64
55	ENST00000317359	ENS T00000317359	ens olfactory receptor, family 10, subfamily AB, member 1 pseudogene [Source:HGNC Symbol;Acc:14804] [ENST00000317359]	2.38	2.17
56	ENST00000366784	ENST00000366784	ens inositol 1,4,5-trisphosphate 3-kinase B [Source:HGNC Symbol;Acc:6179] [ENST00000366784]	2.19	2.58
57	ENST00000446768	ENST00000446768	gb BX117128 Soares_NFL_T_GBC_S1 Homo sapiens cDNA clone IMAGp998H103715, mRNA sequence [BX117128]	2.30	5.98
58	ENST00000481490	ENST00000481490	ens Fibrinogen silencer-binding protein [Source:UniProtKB/Swiss-Prot;Acc:O95073] [ENST00000481490]	2.42	3.88
59	ENST00000521369	ENST00000521369	Homo sapiens similar to solute carrier family 16 (monocarboxylic acid transporters), member 14, mRNA (cDNA clone IMAGE:5726657). [BC040619]	2.26	2.87
60	ENST00000522356	ENST00000522356	Homo sapiens cDNA FLJ38901 fis, clone NT2NE2001040, moderately similar to Homo sapiens GL013 mRNA. [AK096220]	2.13	2.12
61	EPHX2	NM_001979	Homo sapiens epoxide hydrolase 2, cytoplasmic (EPHX2), mRNA [NM_001979]	2.26	2.31
62	ERI2	NM_001142725	Homo sapiens ERI1 exoribonuclease family member 2 (ERI2), transcript variant 1, mRNA [NM_001142725]	3.65	2.05
63	F2RL1	NM_005242	Homo sapiens coagulation factor II (thrombin) receptor-like 1 (F2RL1), mRNA [NM_005242]	3.96	7.92
64	FAM134B	NM_001034850	Homo sapiens family with sequence similarity 134, member B (FAM134B), transcript variant 1, mRNA [NM_001034850]	2.75	2.14
65	FAM153B	NM_001079529	Homo sapiens family with sequence similarity 153, member B (FAM153B), mRNA [NM_001079529]	2.12	2.06
66	FAM19A1	NM_213609	Homo sapiens family with sequence similarity 19 (chemokine (C-C motif)-like), member A1 (FAM19A1), mRNA [NM_213609]	2.11	2.30
67	FAM201A	NR_027294	Homo sapiens family with sequence similarity 201, member A (FAM201A), non-coding RNA [NR_027294]	2.92	4.26
68	FAM26F	NM_001010919	Homo sapiens family with sequence similarity 26, member F (FAM26F), mRNA [NM_001010919]	2.06	2.03
69	FAM43A	NM_153690	Homo sapiens family with sequence similarity 43, member A (FAM43A), mRNA [NM_153690]	2.44	3.18
70	FAM63A	NM_001040217	Homo sapiens family with sequence similarity 63, member A (FAM63A), transcript variant 2, mRNA [NM_001040217]	2.16	2.20
71	FBP1	NM_000507	Homo sapiens fructose-1,6-bisphosphatase 1 (FBP1), transcript variant 1, mRNA [NM_000507]	2.30	2.67
72	FGF9	NM_002010	Homo sapiens fibroblast growth factor 9 (glia-activating factor) (FGF9), mRNA [NM_002010]	2.64	3.64
73	FHIT	NM_002012	Homo sapiens fragile histidine triad gene (FHIT), transcript variant 1, mRNA [NM_002012]	2.49	2.69
74	FLJ36848	AK094167	Homo sapiens cDNA FLJ36848 fis, clone ASTRO2013802. [AK094167]	2.46	3.51
75	FLJ38122	AK095441	Homo sapiens cDNA FLJ38122 fis, clone D3OST3000291. [AK095441]	2.32	2.41
76	FLJ45079	NR_028337	Homo sapiens FLJ45079 protein (FLJ45079), non-coding RNA [NR_028337]	2.36	2.03
77	FOS	NM_005252	Homo sapiens FBJ murine osteosarcoma viral oncogene homolog (FOS), mRNA [NM_005252]	2.22	2.27
78	FSCN1	NM_003088	Homo sapiens fascin homolog 1, actin-bundling protein (Strongylocentrotus purpuratus) (FSCN1), mRNA [NM_003088]	2.37	2.01

79	FZD7	NM_003507	Homo sapiens frizzled homolog 7 (Drosophila) (FZD7), mRNA [NM_003507]	2.66	4.25
80	GADD45A	NM_001924	Homo sapiens growth arrest and DNA-damage-inducible, alpha (GADD45A), transcript variant 1, mRNA [NM_001924]	2.36	2.11
81	GADD45G	NM_006705	Homo sapiens growth arrest and DNA-damage-inducible, gamma (GADD45G), mRNA [NM_006705]	3.31	2.70
82	GEM	NM_005261	Homo sapiens GTP binding protein overexpressed in skeletal muscle (GEM), transcript variant 1, mRNA [NM_005261]	4.97	2.27
83	GJB6	NM_006783	Homo sapiens gap junction protein, beta 6, 30kDa (GJB6), transcript variant 3, mRNA [NM_006783]	2.33	2.72
84	GRASP	NM_181711	Homo sapiens GRP1 (general receptor for phosphoinositides 1)-associated scaffold protein (GRASP), mRNA [NM_181711]	2.74	2.49
85	GRB10	NM_001001555	Homo sapiens growth factor receptor-bound protein 10 (GRB10), transcript variant 4, mRNA [NM_001001555]	2.09	6.06
86	GREM2	NM_022469	Homo sapiens gremlin 2 (GREM2), mRNA [NM_022469]	5.61	6.57
87	HAVCR2	NM_032782	Homo sapiens hepatitis A virus cellular receptor 2 (HAVCR2), mRNA [NM_032782]	2.16	2.15
88	HSBP1L1	NM_001136180	Homo sapiens heat shock factor binding protein 1-like 1 (HSBP1L1), mRNA [NM_001136180]	2.27	2.23
89	HSPA2	NM_021979	Homo sapiens heat shock 70kDa protein 2 (HSPA2), mRNA [NM_021979]	2.38	2.94
90	ID1	NM_002165	Homo sapiens inhibitor of DNA binding 1, dominant negative helix-loop-helix protein (ID1), transcript variant 1, mRNA [NM_002165]	2.78	2.38
91	IGF1R	NM_000875	Homo sapiens insulin-like growth factor 1 receptor (IGF1R), mRNA [NM_000875]	2.96	2.40
92	IL23A	NM_016584	Homo sapiens interleukin 23, alpha subunit p19 (IL23A), mRNA [NM_016584]	3.27	2.23
93	IRS2	NM_003749	Homo sapiens insulin receptor substrate 2 (IRS2), mRNA [NM_003749]	2.38	2.45
94	ISM1	NM_080826	Homo sapiens isthmin 1 homolog (zebrafish) (ISM1), mRNA [NM_080826]	2.98	2.69
95	KANK1	NM_153186	Homo sapiens KN motif and ankyrin repeat domains 1 (KANK1), transcript variant 2, mRNA [NM_153186]	2.70	3.20
96	KCNQ1	NM_000218	Homo sapiens potassium voltage-gated channel, KQT-like subfamily, member 1 (KCNQ1), transcript variant 1, mRNA [NM_000218]	2.69	2.41
97	KCTD12	NM_138444	Homo sapiens potassium channel tetramerisation domain containing 12 (KCTD12), mRNA [NM_138444]	3.59	3.66
98	KCTD3	NM_016121	Homo sapiens potassium channel tetramerisation domain containing 3 (KCTD3), mRNA [NM_016121]	2.30	7.25
99	KLHDC9	NM_001007255	Homo sapiens kelch domain containing 9 (KLHDC9), transcript variant 2, mRNA [NM_001007255]	2.08	2.12
100	KLHL34	NM_153270	Homo sapiens kelch-like 34 (Drosophila) (KLHL34), mRNA [NM_153270]	2.39	2.46
101	LASS6	NM_203463	Homo sapiens LAG1 homolog, ceramide synthase 6 (LASS6), mRNA [NM_203463]	2.11	2.05
102	LAYN	NM_178834	Homo sapiens layilin (LAYN), mRNA [NM_178834]	2.63	6.24
103	LDOC1	NM_012317	Homo sapiens leucine zipper, down-regulated in cancer 1 (LDOC1), mRNA [NM_012317]	2.90	2.20
104	LEF1	NM_016269	Homo sapiens lymphoid enhancer-binding factor 1 (LEF1), transcript variant 1, mRNA [NM_016269]	2.22	2.09
105	LMF1	NR_036442	Homo sapiens lipase maturation factor 1 (LMF1), transcript variant 4, non-coding RNA [NR_036442]	2.90	2.98
106	LOC100128252	NR_036522	Homo sapiens hypothetical LOC100128252 (LOC100128252), transcript variant 2, non-coding RNA [NR_036522]	2.22	3.00
107	LOC100131662	AK127156	Homo sapiens cDNA FLJ45218 fis, clone BRCAN2019653. [AK127156]	2.42	2.02
108	LOC283028	AK092783	Homo sapiens cDNA FLJ35464 fis, clone SMINT2005402. [AK092783]	2.01	2.28
109	LOC645586	AK057937	Homo sapiens cDNA FLJ25208 fis, clone REC05984. [AK057937]	2.76	2.43
110	LOH12CR2	NR_024061	Homo sapiens loss of heterozygosity, 12, chromosomal region 2 (LOH12CR2), non-coding RNA [NR_024061]	2.52	2.07
111	LRRN3	NM_018334	Homo sapiens leucine rich repeat neuronal 3 (LRRN3), transcript variant 3, mRNA [NM_018334]	2.56	2.60
112	LYPD3	NM_014400	Homo sapiens LY6/PLAUR domain containing 3 (LYPD3), mRNA [NM_014400]	2.69	4.09
113	LZTS1	NM_021020	Homo sapiens leucine zipper, putative tumor suppressor 1 (LZTS1), mRNA [NM_021020]	2.95	3.51
114	MAL	NM_002371	Homo sapiens mal, T-cell differentiation protein (MAL), transcript variant a, mRNA [NM_002371]	2.60	2.45
115	MARVELD1	NM_031484	Homo sapiens MARVEL domain containing 1 (MARVELD1), mRNA [NM_031484]	2.06	2.19
116	MEST	NM_002402	Homo sapiens mesoderm specific transcript homolog (mouse) (MEST), transcript variant 1, mRNA [NM_002402]	2.91	3.02
117	MYC	NM_002467	Homo sapiens v-myc myelocytomatosis viral oncogene homolog (avian) (MYC), mRNA [NM_002467]	3.20	2.25
118	NAP1L3	NM_004538	Homo sapiens nucleosome assembly protein 1-like 3 (NAP1L3), mRNA [NM_004538]	2.47	10.52

119	NBEA	NM_015678	Homo sapiens neurobeachin (NBEA), mRNA [NM_015678]	2.74	2.79
120	NBL1	NM_182744	Homo sapiens neuroblastoma, suppression of tumorigenicity 1 (NBL1), transcript variant 1, mRNA [NM_182744]	3.12	2.59
121	NCRNA00087	NR_024493	Homo sapiens non-protein coding RNA 87 (NCRNA00087), non-coding RNA [NR_024493]	2.27	2.05
122	NEFL	NM_006158	Homo sapiens neurofilament, light polypeptide (NEFL), mRNA [NM_006158]	2.90	3.19
123	NEFM	NM_005382	Homo sapiens neurofilament, medium polypeptide (NEFM), transcript variant 1, mRNA [NM_005382]	5.39	8.17
124	NELL2	NM_006159	Homo sapiens NEL-like 2 (chicken) (NELL2), transcript variant 2, mRNA [NM_006159]	2.45	2.84
125	NET1	NM_001047160	Homo sapiens neuroepithelial cell transforming 1 (NET1), transcript variant 1, mRNA [NM_001047160]	2.47	2.19
126	NGFRAP1	NM_014380	Homo sapiens nerve growth factor receptor (TNFRSF16) associated protein 1 (NGFRAP1), transcript variant 3, mRNA [NM_014380]	2.16	2.45
127	NKX3-1	NM_006167	Homo sapiens NK3 homeobox 1 (NKX3-1), mRNA [NM_006167]	3.93	3.72
128	NOG	NM_005450	Homo sapiens noggin (NOG), mRNA [NM_005450]	3.05	3.75
129	NRARP	NM_001004354	Homo sapiens NOTCH-regulated ankyrin repeat protein (NRARP), mRNA [NM_001004354]	3.18	3.11
130	NRCAM	NM_001037132	Homo sapiens neuronal cell adhesion molecule (NRCAM), transcript variant 1, mRNA [NM_001037132]	2.28	3.22
131	NT5E	NM_002526	Homo sapiens 5'-nucleotidase, ecto (CD73) (NT5E), mRNA [NM_002526]	2.06	4.14
132	PCSK5	NM_006200	Homo sapiens proprotein convertase subtilisin/kexin type 5 (PCSK5), transcript variant 2, mRNA [NM_006200]	3.42	2.82
133	PHLDA1	NM_007350	Homo sapiens pleckstrin homology-like domain, family A, member 1 (PHLDA1), mRNA [NM_007350]	2.33	2.58
134	PHLDA2	NM_003311	Homo sapiens pleckstrin homology-like domain, family A, member 2 (PHLDA2), mRNA [NM_003311]	2.14	2.46
135	PLAG1	NM_002655	Homo sapiens pleiomorphic adenoma gene 1 (PLAG1), transcript variant 1, mRNA [NM_002655]	3.76	2.82
136	PLK2	NM_006622	Homo sapiens polo-like kinase 2 (PLK2), mRNA [NM_006622]	4.79	4.77
137	PTPN13	NM_080685	Homo sapiens protein tyrosine phosphatase, non-receptor type 13 (APO-1/CD95 (Fas)-associated phosphatase) (PTPN13), transcript variant 4, mRNA [NM_080685]	2.57	3.93
138	PTPN20B	NM_001042357	Homo sapiens protein tyrosine phosphatase, non-receptor type 20B (PTPN20B), transcript variant 1, mRNA [NM_001042357]	2.23	2.41
139	RASD1	NM_016084	Homo sapiens RAS, dexamethasone-induced 1 (RASD1), transcript variant 1, mRNA [NM_016084]	2.43	2.98
140	RASL11B	NM_023940	Homo sapiens RAS-like, family 11, member B (RASL11B), mRNA [NM_023940]	2.47	3.80
141	RBM11	NM_144770	Homo sapiens RNA binding motif protein 11 (RBM11), mRNA [NM_144770]	2.76	2.58
142	RCVRN	NM_002903	Homo sapiens recoverin (RCVRN), mRNA [NM_002903]	2.12	2.26
143	RGS16	NM_002928	Homo sapiens regulator of G-protein signaling 16 (RGS16), mRNA [NM_002928]	2.62	2.16
144	RIN1	NM_004292	Homo sapiens Ras and Rab interactor 1 (RIN1), mRNA [NM_004292]	2.83	2.17
145	RRAGD	NM_021244	Homo sapiens Ras-related GTP binding D (RRAGD), mRNA [NM_021244]	3.03	5.49
146	S100B	NM_006272	Homo sapiens S100 calcium binding protein B (S100B), mRNA [NM_006272]	4.08	3.97
147	SCML1	NM_001037540	Homo sapiens sex comb on midleg-like 1 (Drosophila) (SCML1), transcript variant 1, mRNA [NM_001037540]	2.10	2.39
148	SFRP5	NM_003015	Homo sapiens secreted frizzled-related protein 5 (SFRP5), mRNA [NM_003015]	2.13	3.20
149	SGK1	NM_005627	Homo sapiens serum/glucocorticoid regulated kinase 1 (SGK1), transcript variant 1, mRNA [NM_005627]	3.06	2.83
150	SH3YL1	NM_015677	Homo sapiens SH3 domain containing, Ysc84-like 1 (S. cerevisiae) (SH3YL1), transcript variant 1, mRNA [NM_015677]	2.33	2.56
151	SLC16A10	NM_018593	Homo sapiens solute carrier family 16, member 10 (aromatic amino acid transporter) (SLC16A10), mRNA [NM_018593]	3.70	3.67
152	SLC40A1	NM_014585	Homo sapiens solute carrier family 40 (iron-regulated transporter), member 1 (SLC40A1), mRNA [NM_014585]	3.88	6.01
153	SNAI1	NM_005985	Homo sapiens snail homolog 1 (Drosophila) (SNAI1), mRNA [NM_005985]	2.69	2.79
154	SNN	NM_003498	Homo sapiens stannin (SNN), mRNA [NM_003498]	2.96	2.69
155	SOAT2	NM_003578	Homo sapiens sterol O-acyltransferase 2 (SOAT2), mRNA [NM_003578]	2.15	2.63
156	SOCS3	NM_003955	Homo sapiens suppressor of cytokine signaling 3 (SOCS3), mRNA [NM_003955]	2.34	2.82
157	SOX8	NM_014587	Homo sapiens SRY (sex determining region Y)-box 8 (SOX8), mRNA [NM_014587]	2.02	2.28
158	SPG20	NM_015087	Homo sapiens spastic paraplegia 20 (Troyer syndrome) (SPG20), transcript variant 1, mRNA [NM_015087]	3.05	5.10

159	SPINK2	NM_021114	Homo sapiens serine peptidase inhibitor, Kazal type 2 (acrosin-trypsin inhibitor) (SPINK2), mRNA [NM_021114]	3.56	3.66
160	SPINT2	NM_021102	Homo sapiens serine peptidase inhibitor, Kunitz type, 2 (SPINT2), transcript variant a, mRNA [NM_021102]	2.50	2.74
161	TAF4B	NM_005640	Homo sapiens TAF4b RNA polymerase II, TATA box binding protein (TBP)-associated factor, 105kDa (TAF4B), mRNA [NM_005640]	2.62	2.35
162	TCEA3	NM_003196	Homo sapiens transcription elongation factor A (SII), 3 (TCEA3), mRNA [NM_003196]	2.48	2.94
163	TCEAL2	NM_080390	Homo sapiens transcription elongation factor A (SII)-like 2 (TCEAL2), mRNA [NM_080390]	3.52	3.09
164	TIMD4	NM_138379	Homo sapiens T-cell immunoglobulin and mucin domain containing 4 (TIMD4), transcript variant 1, mRNA [NM_138379]	2.16	3.17
165	TPST1	NM_003596	Homo sapiens tyrosylprotein sulfotransferase 1 (TPST1), mRNA [NM_003596]	2.93	3.37
166	VIPR1	NM_004624	Homo sapiens vasoactive intestinal peptide receptor 1 (VIPR1), mRNA [NM_004624]	2.28	3.07
167	WNT7A	NM_004625	Homo sapiens wingless-type MMTV integration site family, member 7A (WNT7A), mRNA [NM_004625]	2.83	5.18
168	WNT7B	NM_058238	Homo sapiens wingless-type MMTV integration site family, member 7B (WNT7B), mRNA [NM_058238]	2.74	3.43
169	X57723	X57723	gb H.sapiens rearranged TCR Vbeta 16a mRNA for T cell receptor. [X57723]	2.09	2.45
170	ZBED3	NM_032367	Homo sapiens zinc finger, BED-type containing 3 (ZBED3), mRNA [NM_032367]	2.29	2.18
171	ZNF165	NM_003447	Homo sapiens zinc finger protein 165 (ZNF165), mRNA [NM_003447]	2.94	2.72
172	ZNF662	NM_207404	Homo sapiens zinc finger protein 662 (ZNF662), transcript variant 1, mRNA [NM_207404]	2.62	2.18
173	ZNF703	NM_025069	Homo sapiens zinc finger protein 703 (ZNF703), mRNA [NM_025069]	3.05	3.72

The number of upregulated genes was 173. Pale pink labels show 64 membrane protein-related genes and 7 transcription factors. Dark pink labels show the top 30 genes. Yellow labels show 7 genes upregulated in CD8+ALDHhigh cells as determined by RT-PCR analysis.
