# Supplementary material for: Identification of a novel human memory T-cell population with the characteristics of stem-like chemo-resistance
Source: Oncoimmunology. 2016 Jun 8;5(6):e1165376. doi: 10.1080/2162402X.2016.1165376 (PMC4938359; doi:10.1080/2162402X.2016.1165376)
Supplement: KONI_A_1165376_supplemental_material.zip [file koni-05-06-1165376-s001.zip › KONI_A_1165376_s02 legends.docx]

**Supplemental Figure legends**

**Figure S1. CD8^+^ALDH^high^ T cells in PBMC contained a population with the characteristics of drug-resistance and responsibility for TCR stimulation.**

(A) Post-sort analysis of purified ALDH^high^ and ALDH^low^ cells.

(B) The mRNA expression of ALDH1A1 in CD8^+^ALDH^high^ and CD8^+^ALDH^low^ cells. Data represent mean ± SD. Statistically significant differences were determined with the Mann–Whitney *U* test.

(C) The microscopic features of CD8^+^ALDH^high^ and CD8^+^ALDH^low^ cells reacted with anti-CD3/CD28 microbeads. Data are representative of three independent experiments.

(D) CD8^+^ALDH^high/low^ cells were cultured in the presence of serially diluted adriamycin and labeled with Annexin V.

(E) Representative FACS plots of CD45RA and CD62L expression in CD8^+^ALDH^high^ and CD8^+^ALDH^low^ cells.

(F) Proportions of ALDH^high^ and ALDH^low^ cells in CD8^+^ T cell subsets from adult peripheral blood (n=9). Each point represents data from an individual healthy donor, and bars represent means. Statistically significant differences were determined with the Mann–Whitney *U* test.

(G) CD8^+^ALDH^high^ or CD8^+^ALDH^low^ cells were activated with bCD3/CD28 and cultured with IL-7 and IL-15 for 6-7 days, and then analyzed for expression of CD45RA and CD62L by flow cytometry (left panel). Cell numbers of CD8^+^ T cell subsets generated from CD8^+^ALDH^high^ and CD8^+^ALDH^low^ cells stimulated with bCD3/CD28, IL-7 and IL-15 (right panel). Data are representative of three independent experiments.

**Figure S2. Fluorescence minus one (FMO) control in human T cells.**

CD45RA, CD62L, CXCR3, CD73 FMO controls in TCR-stimulated T cells.

**Figure S3. The characteristics of T_YM_ excluding T_SCM_  (CD95+) cells were comparable to those of T_YM_ including CD95^+^ cells.**

(A) Expansion of T_YM_, T_YM_ (CD95^-^) cells and known memory CD8^+^ T cell subsets activated with bCD3/CD28 and cultured with IL-7 and IL-15 at days 6-7. Data represent mean ± SD of five independent experiments. Statistically significant differences were determined with the Mann–Whitney *U* test.

(B) T_YM_, T_YM_ (CD95^-^), T_CM_ and T_EM_ cells were cultured in the presence of serially diluted carboplatin, and labeled with Annexin V.

(C) Differentiation of T-cell subsets including T_YM_ (CD95^-^) upon TCR stimulation using bCD3/CD28, IL-7 and IL-15 at Day 7. Data are representative of three independent experiments.

(D) T_N_, T_YM_, T_YM_ (CD95^-^), T_CM_ and T_EM_ cells were stimulated with CD8^-^ T cells pulsed with epitopes from EBV and HIV, and cultured for 12-14 days in the presence of IL-2 and IL-7. The percentage of tetramer^+^ events is shown.

(E) Detection of CD107a exposed on the cell surface after antigen stimulation. HLA-A*24:02-restricted CTL were stimulated using an epitope peptide in the presence of an FITC-labeled CD107a monoclonal antibody and cultured for 5 hours at 37°C. After culture, the cells were stained with HLA-A*24:02 EBV tetramer-PE. T_YM_ (CD95^-^) cells were defined by CD73^+^CD45RA^+^CD62L^+^CXCR3^+^CD95^-^.
