# Supplementary material for: Identification of a novel human memory T-cell population with the characteristics of stem-like chemo-resistance
Source: Oncoimmunology. 2016 Jun 8;5(6):e1165376. doi: 10.1080/2162402X.2016.1165376 (PMC4938359; doi:10.1080/2162402X.2016.1165376)
Supplement: KONI_A_1165376_supplemental_material.zip [file koni-05-06-1165376-s001.zip › KONI_A_1165376_s02.pptx]

## Slide 1
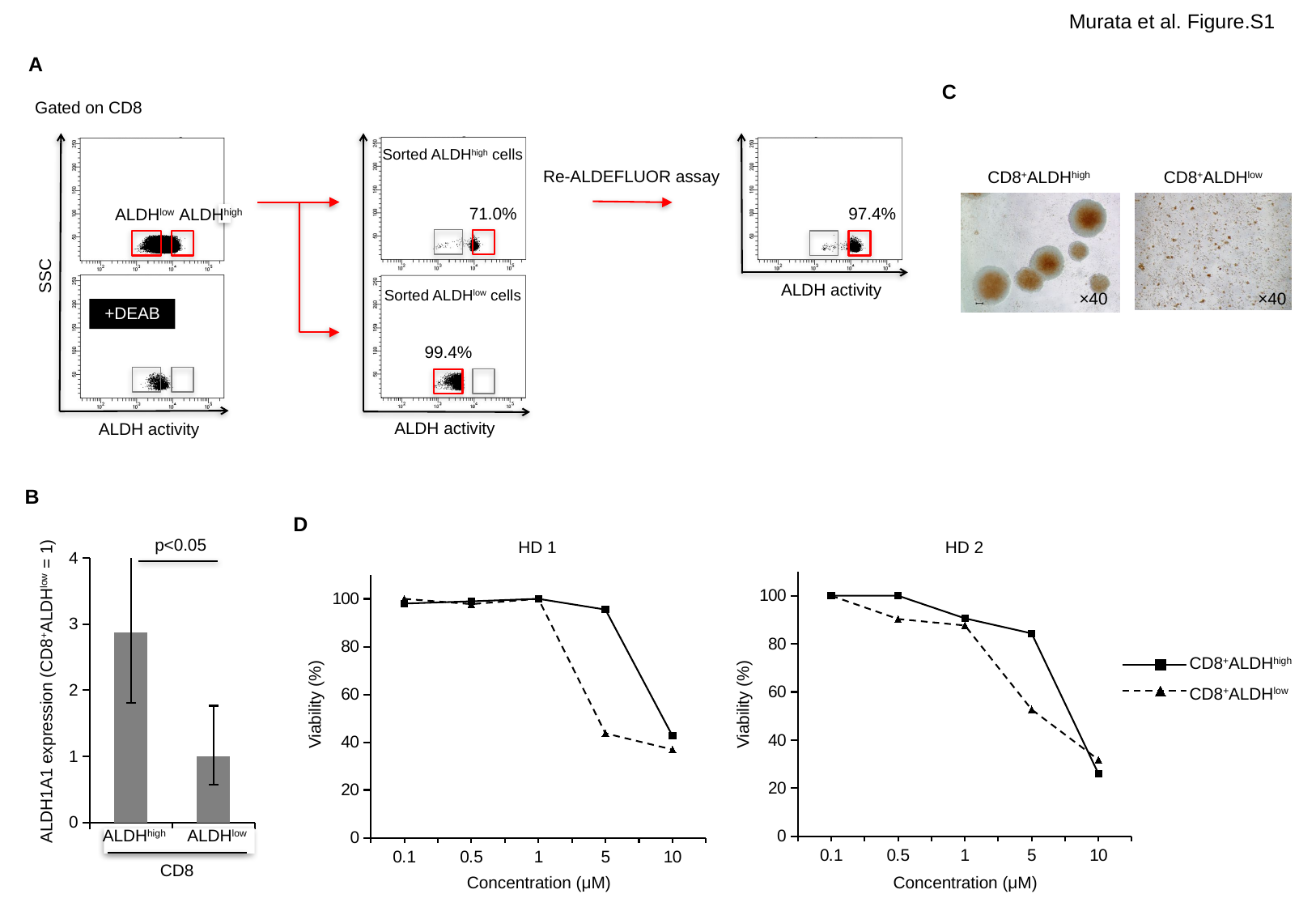

Murata et al. Figure.S1
A
C
Gated on CD8
Sorted ALDHhigh cells
CD8+ALDHhigh
CD8+ALDHlow
Re-ALDEFLUOR assay
97.4%
71.0%
ALDHhigh
ALDHlow
SSC
ALDH activity
×40
×40
Sorted ALDHlow cells
+DEAB
99.4%
ALDH activity
ALDH activity
B
D
### Chart
| Category | ALDH high | ALDH low |
|---|---|---|
| 0.1 | 100.0 | 100.0 |
| 0.5 | 100.0 | 90.30100334448115 |
| 1 | 90.58971141781655 | 87.62541806020045 |
| 5 | 84.31618569636134 | 52.67558528428094 |
| 10 | 25.97239648682558 | 31.77257525083612 |
### Chart
| Category | ALDH high | ALDH low |
|---|---|---|
| 0.1 | 98.0146290491118 | 100.0 |
| 0.5 | 98.95506792058517 | 97.77777777777735 |
| 1 | 100.0 | 100.0 |
| 5 | 95.50679205851557 | 43.7037037037037 |
| 10 | 42.6332288401254 | 36.91358024691358 | p<0.05
HD 1
HD 2
### Chart
| Category | |
|---|---|
| high | 2.876727819442749 |
| low | 1.0 |CD8+ALDHhigh
ALDH1A1 expression (CD8+ALDHlow = 1)
CD8+ALDHlow
Viability (%)
Viability (%)
ALDHlow
ALDHhigh
CD8
Concentration (μM)
Concentration (μM)

## Slide 2
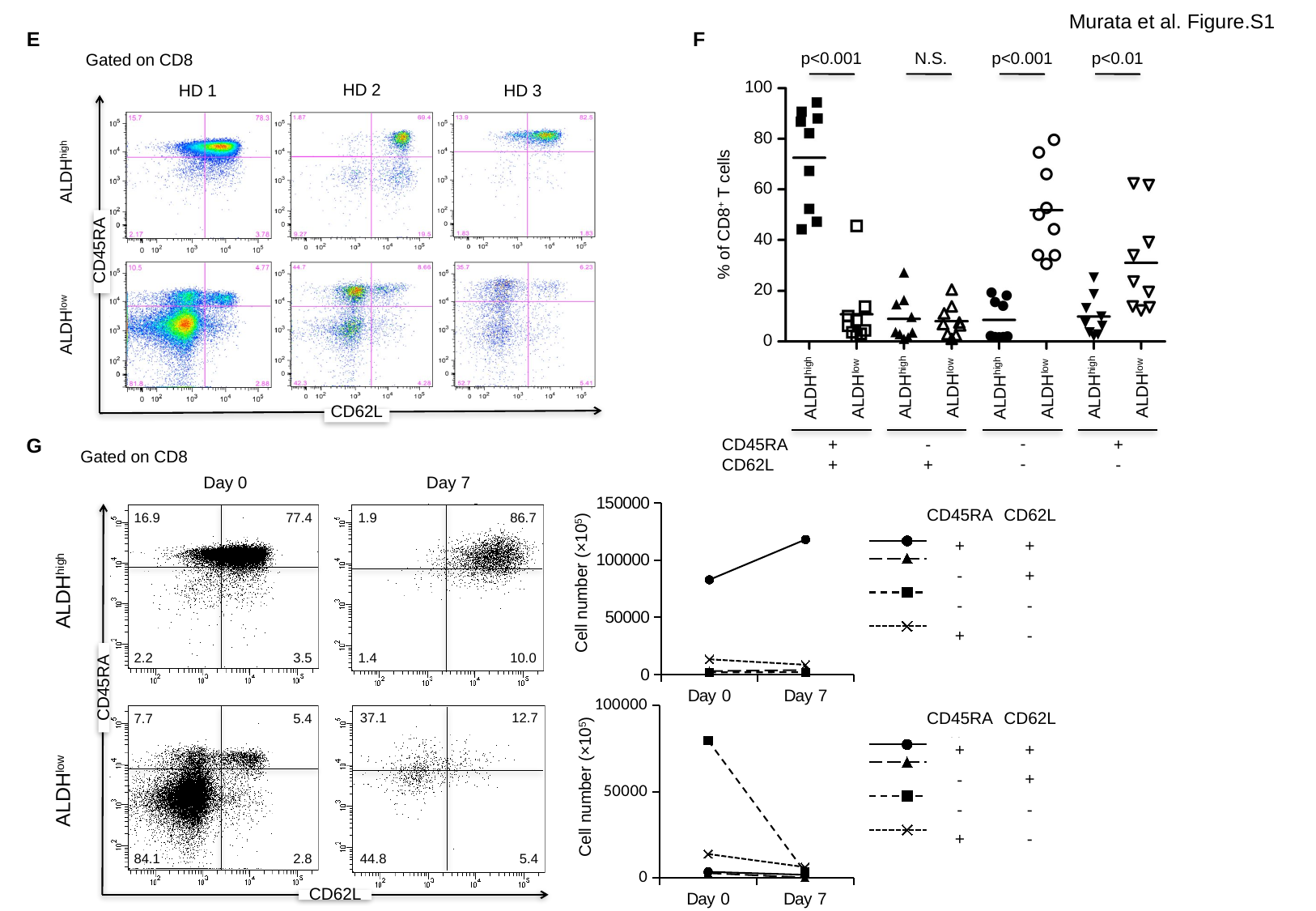

Murata et al. Figure.S1
E
F
p<0.001
N.S.
p<0.001
p<0.01
Gated on CD8
100
HD 2
HD 1
HD 3
80
ALDHhigh
60
% of CD8+ T cells
40
CD45RA
20
ALDHlow
0
ALDHhigh
ALDHhigh
ALDHlow
ALDHlow
ALDHhigh
ALDHhigh
ALDHlow
ALDHlow
CD62L
G
-
-
+
-
CD45RA
CD62L
+
+
-
+
Gated on CD8
Day 0
Day 7
### Chart
| Category | N | CM | EM | Ef |
|---|---|---|---|---|
| Day 0 | 82800.0 | 2900.0 | 1800.0 | 13200.0 |
| Day 7 | 118008.0 | 3828.0 | 1848.0 | 8448.0 |CD62L
CD45RA
16.9
77.4
1.9
86.7
+
+
+
-
Cell number (×105)
ALDHhigh
-
-
-
+
2.2
3.5
1.4
10.0
CD45RA
### Chart
| Category | N | CM | EM | Ef |
|---|---|---|---|---|
| Day 0 | 3600.0 | 2900.0 | 79599.99999999997 | 13900.0 |
| Day 7 | 1896.0 | 252.0 | 3516.0 | 6336.0 |CD62L
CD45RA
37.1
12.7
7.7
5.4
+
+
+
-
Cell number (×105)
ALDHlow
-
-
-
+
44.8
5.4
84.1
2.8
CD62L

## Slide 3
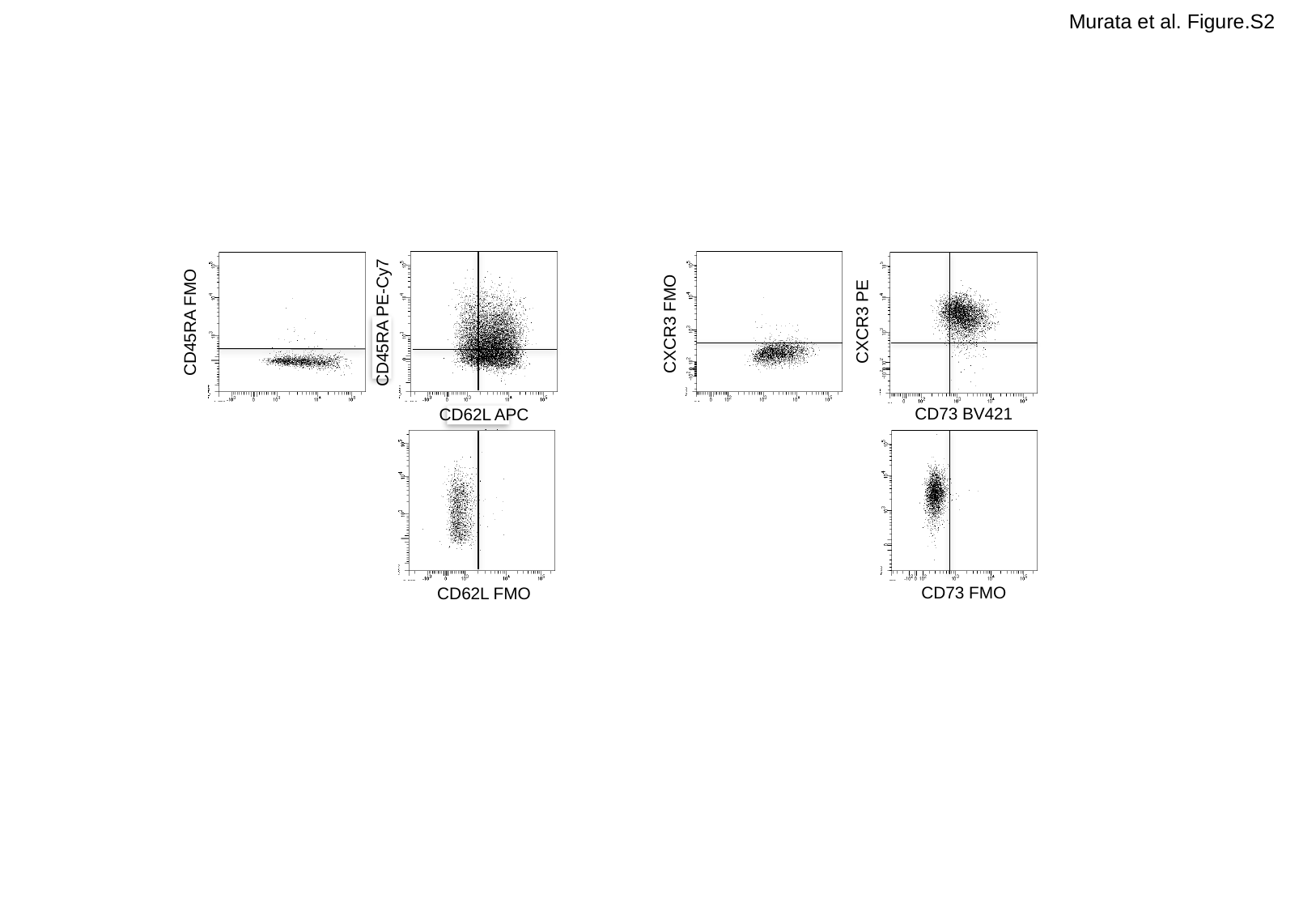

Murata et al. Figure.S2
CXCR3 PE
CD45RA FMO
CD45RA PE-Cy7
CXCR3 FMO
CD73 BV421
CD62L APC
CD73 FMO
CD62L FMO

## Slide 4
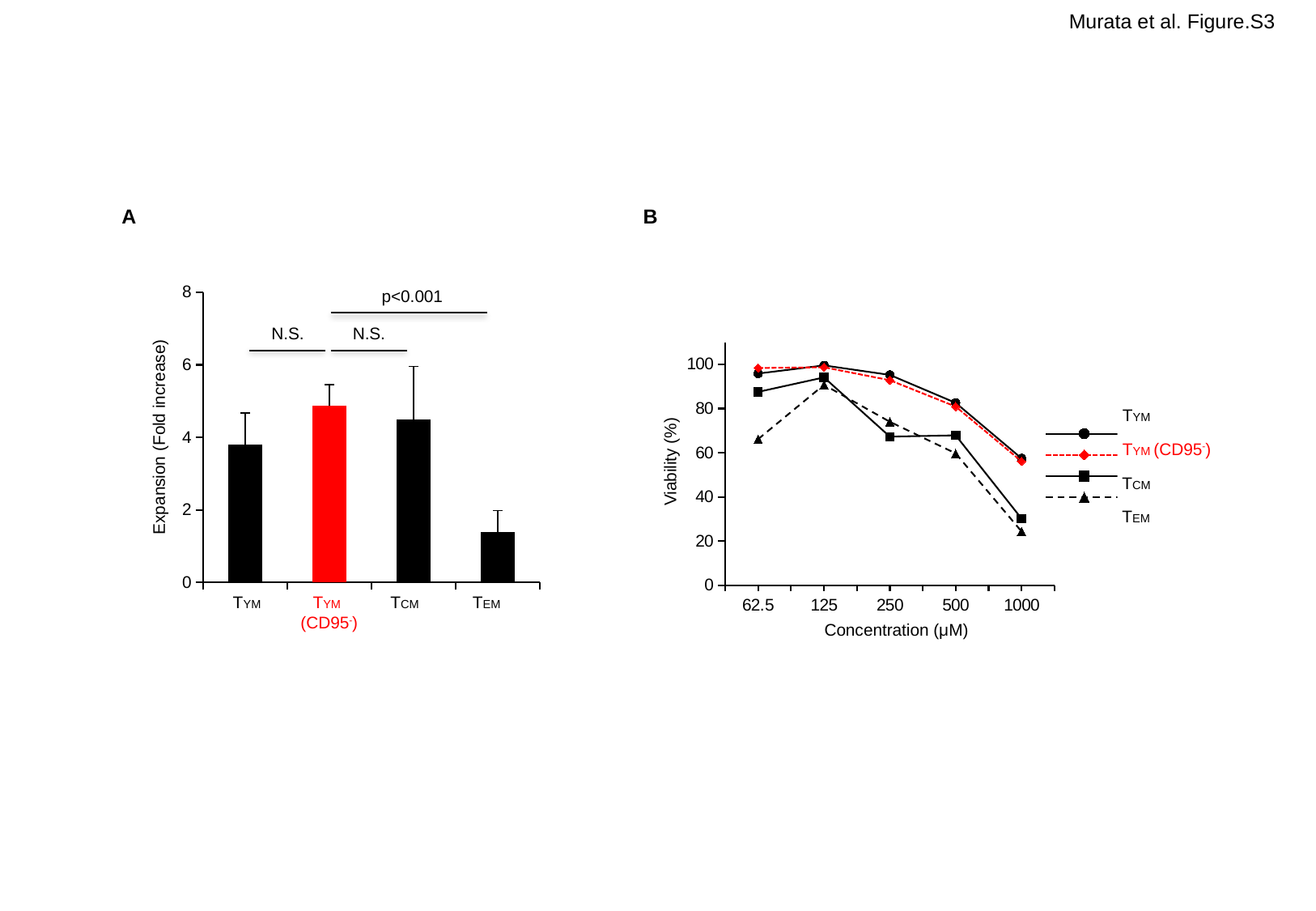

Murata et al. Figure.S3
A
B
### Chart
| Category | |
|---|---|
| Tym | 3.799333333333333 |
| Tym (CD95-) | 4.875 |
| Tcm | 4.488801298327825 |
| Tem | 1.395375 | p<0.001
 N.S.
 N.S.
### Chart
| Category | Tym | Tym CD95- | Tcm | Tem |
|---|---|---|---|---|
| 62.5 | 95.79831932773108 | 98.32810867293566 | 87.5 | 66.14285714285613 |
| 125 | 99.47478991596638 | 98.64158829676055 | 94.0476190476192 | 90.71428571428572 |
| 250 | 95.16806722689019 | 92.78996865203762 | 67.2619047619049 | 74.0 |
| 500 | 82.56302521008398 | 80.87774294670783 | 67.85714285714278 | 59.71428571428572 |
| 1000 | 57.45798319327683 | 56.11285266457681 | 30.23809523809523 | 24.42857142857142 |TYM
Expansion (Fold increase)
TYM (CD95-)
Viability (%)
TCM
TEM
TYM
TYM
 (CD95-)
TCM
TEM
Concentration (μM)

## Slide 5
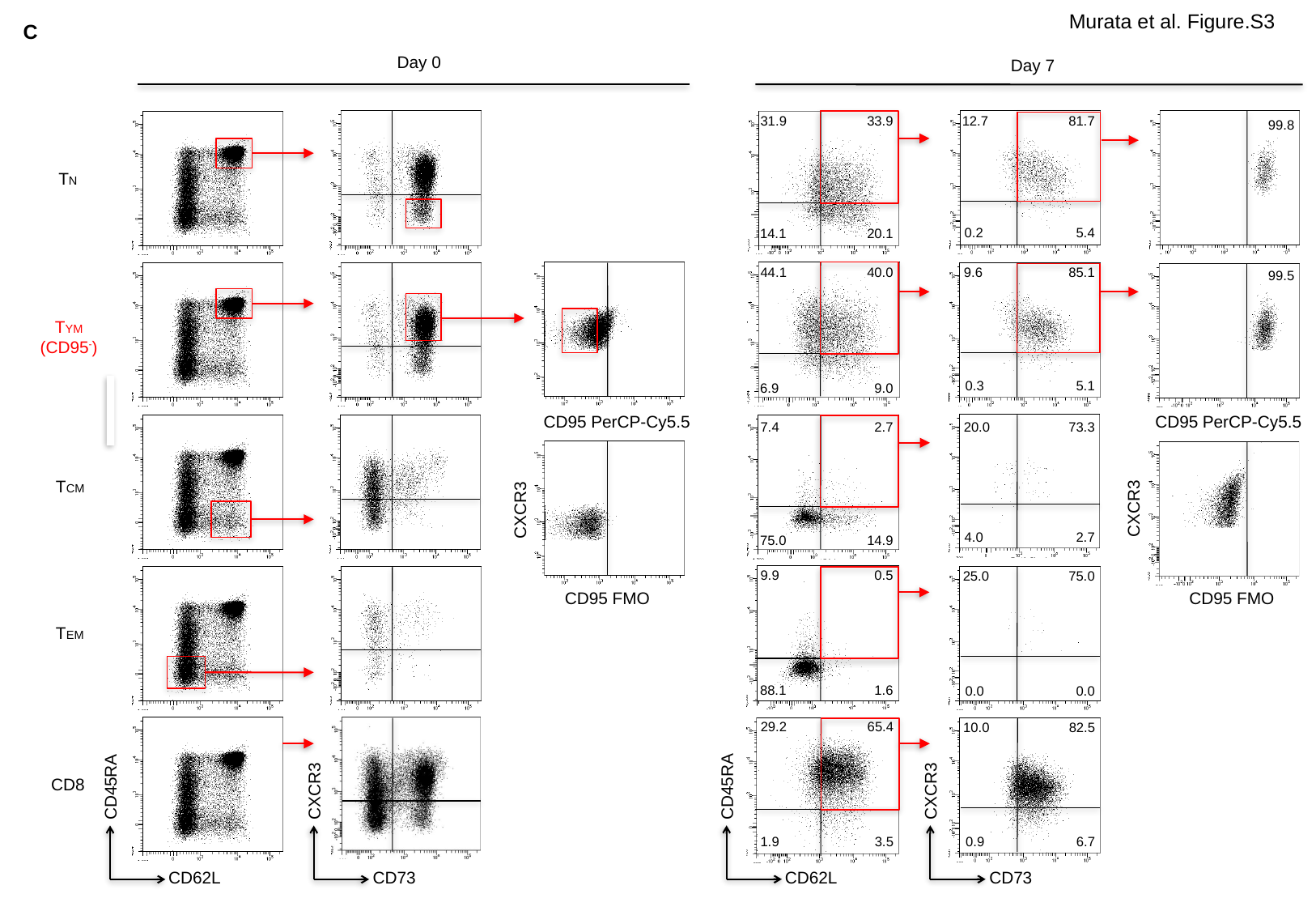

Murata et al. Figure.S3
C
Day 0
Day 7
31.9
33.9
12.7
81.7
99.8
TN
0.2
5.4
14.1
20.1
44.1
40.0
9.6
85.1
99.5
TYM
(CD95-)
0.3
5.1
6.9
9.0
CD95 PerCP-Cy5.5
CD95 PerCP-Cy5.5
7.4
2.7
20.0
73.3
 TCM
CXCR3
CXCR3
4.0
2.7
75.0
14.9
9.9
0.5
25.0
75.0
CD95 FMO
CD95 FMO
 TEM
88.1
1.6
0.0
0.0
29.2
65.4
10.0
82.5
CD8
CD45RA
CD45RA
CXCR3
CXCR3
1.9
3.5
0.9
6.7
CD62L
CD73
CD62L
CD73

## Slide 6
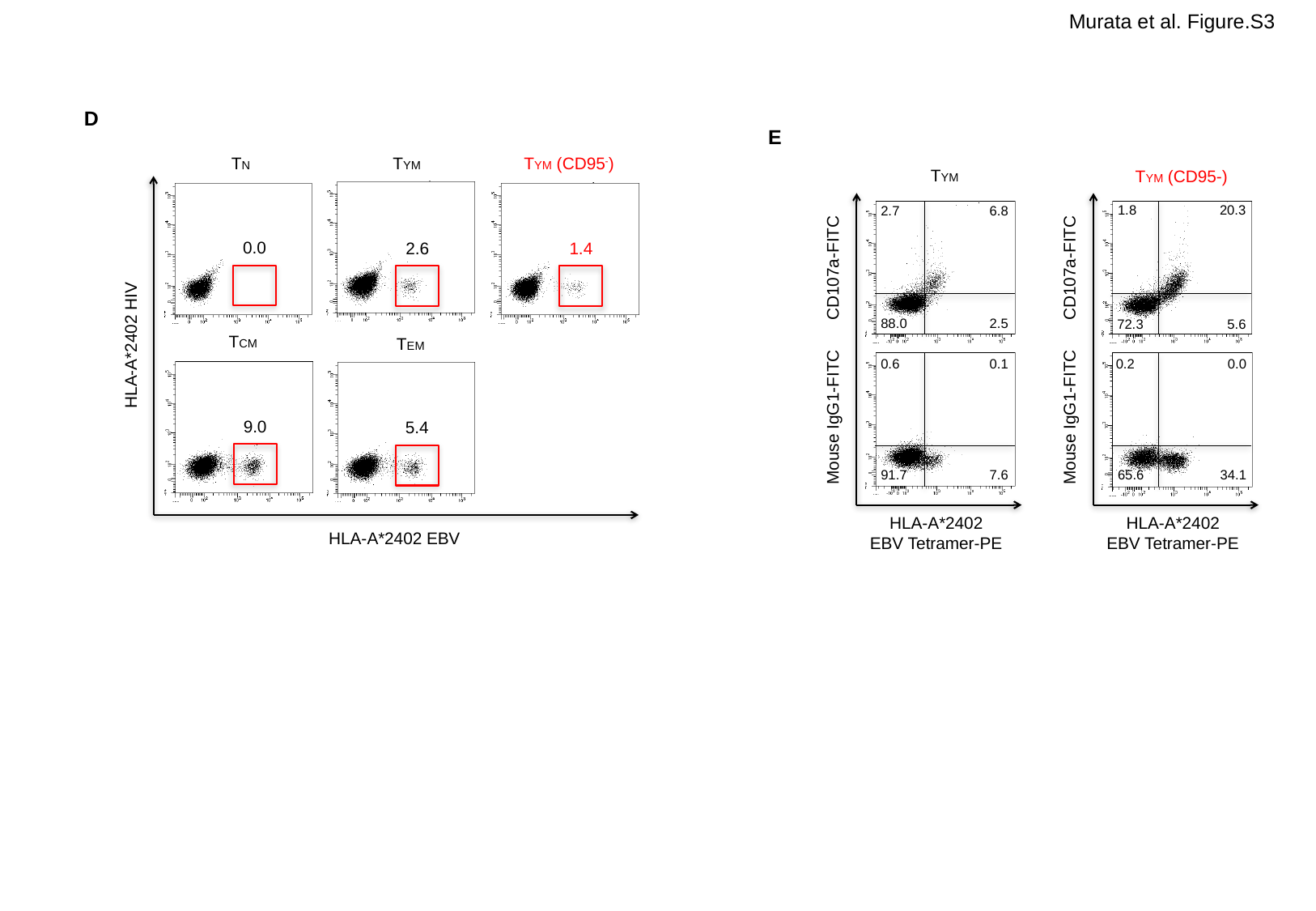

Murata et al. Figure.S3
D
E
TYM (CD95-)
TN
TYM
TYM
TYM (CD95-)
2.6
0.0
1.4
1.8
20.3
2.7
6.8
CD107a-FITC
CD107a-FITC
88.0
2.5
72.3
5.6
 TCM
 TEM
HLA-A*2402 HIV
0.6
0.1
0.2
0.0
5.4
9.0
Mouse IgG1-FITC
Mouse IgG1-FITC
91.7
65.6
34.1
7.6
HLA-A*2402
EBV Tetramer-PE
HLA-A*2402
EBV Tetramer-PE
HLA-A*2402 EBV
